# Fossil evidence reveals how plants responded to cooling during the Cretaceous-Paleogene transition

**DOI:** 10.1186/s12870-019-1980-y

**Published:** 2019-09-13

**Authors:** Yi-Ming Cui, Wei Wang, David K. Ferguson, Jian Yang, Yu-Fei Wang

**Affiliations:** 1 State Key Laboratory of Systematic and Evolutionary Botany, Institute of Botany, Chinese Academy of Sciences, Beijing, 100093 China; 2 Department of Palaeontology, University of Vienna, Althanstrasse 14, A-1090 Vienna, Austria; 3 State Key Laboratory of Palaeobiology and Stratigraphy, Nanjing Institute of Geology and Palaeontology, Chinese Academy of Sciences, Nanjing, 210008 China; 4 University of Chinese Academy of Sciences, Beijing, 100049 China

**Keywords:** Adaptation, Climate change, K-Pg transition, Fossil plant, Morphology, *Mesocyparis*

## Abstract

**Background:**

Around the Cretaceous-Paleogene (K-Pg) boundary, an obvious global cooling occurred, which resulted in dramatic changes in terrestrial ecosystems and the evolutionary trends of numerous organisms. However, how plant lineages responded to the cooling has remained unknown until now. Between *ca*. 70–60 Ma *Mesocyparis* McIver & Basinger (Cupressaceae), an extinct conifer genus, was distributed from eastern Asia to western North America and provides an excellent opportunity to solve this riddle.

**Results:**

Here we report a new species, *Mesocyparis sinica* from the early Paleocene of Jiayin, Heilongjiang, northeastern China. By integrating lines of evidence from phylogeny and comparative morphology of *Mesocyparis*, we found that during ca.70–60 Ma, the size of seed cone of *Mesocyparis* more than doubled, probably driven by the cooling during the K-Pg transition, which might be an effective adaptation for seed dispersal by animals. More importantly, we discovered that the northern limit of this genus, as well as those of two other arboreal taxa *Metasequoia* Miki ex Hu et Cheng (gymnosperm) and *Nordenskioldia* Heer (angiosperm), migrated ca.4–5° southward in paleolatitude during this time interval.

**Conclusions:**

Our results suggest that the cooling during the K-Pg transition may have been responsible for the increase in size of the seed cone of *Mesocyparis* and have driven the migration of plants southwards.

**Electronic supplementary material:**

The online version of this article (10.1186/s12870-019-1980-y) contains supplementary material, which is available to authorized users.

## Background

The cooling from the Maastrichtian to the Paleocene that occurred during the Cretaceous-Paleogene (K-Pg) transition (ca. 70–60 Ma), has been recorded by land plant leaf data [1] and marine oxygen isotope records [2]. These dramatic environmental and climate changes [3–5] altered the composition of terrestrial ecosystems and the evolutionary trends of numerous organisms. Examples include the extinction of non-avian dinosaurs and the rise of mammals, the decline of gymnosperms, and the upsurge of angiosperms [6–8]. Some zoological cases indicated how animal taxa changed their morphological features during the K-Pg transition [8–10]. For instance, a previous study found that the maximum body size and dental complexity disparity of multituberculate mammals across the K-Pg boundary was associated with dietary expansion [8]. On the other hand, botanical evidence revealed regional vegetation change during this time interval [11, 12] and provided estimates of paleotemperature in both hemispheres [2, 12–14]. Leaf physiognomic analysis showed that the 13 °C mean annual temperature isotherm (mesothermal-microthemal boundary) of high latitudinal western North America moved southwards from ca. 65–75 °N in the Maastrichtian to ca. 55 °N in the Danian [15, 16]. Besides, a study focused on the range changes of *Metasequoia* around the world since the Late Cretaceous has shown the knowledge of the distributional changes of this genus across the K-Pg transition [17]. Nevertheless, it has remained relatively unknown how specific plant taxa responded to the cooling during the K-Pg transition.

The extinct genus *Mesocyparis* McIver & Basinger (Cupressaceae) was distributed in eastern Asia and western North America from the Late Cretaceous to the Paleocene, *ca*. 70–60 Ma [18–22]. Here, we report new fossil material of this genus from the upper part of the Wuyun Formation, Jiayin County, Heilongjiang Province. The new *Mesocyparis* fossils from Northeast China, together with the other four species of this genus in the Northern Hemisphere, provide us with an opportunity to evaluate adaptions within the genus.

## Results

### Systematics

**Order:** Coniferales.

**Family:** Cupressaceae.

**Subfamily:** Cupressoideae.

**Genus:** *Mesocyparis* McIver et Basinger.

**Species:** ***Mesocyparis sinica*** Y. M. Cui, W. Wang, D. K. Ferguson, J. Yang et Y. F. Wang, **sp. nov.**

**Fig. 1:**
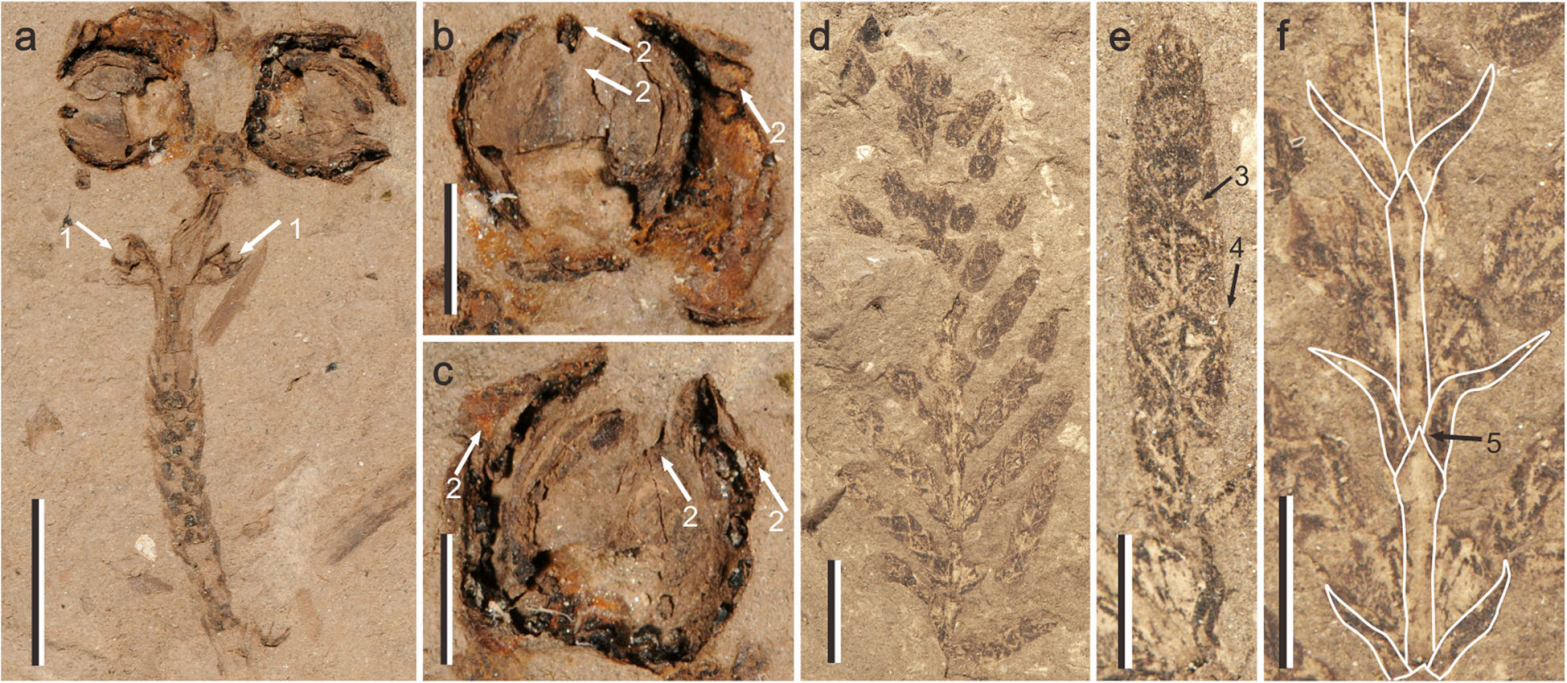
Morphology of *Mesocyparis sini*ca. (**a**)-(**c**) fertile shoot, specimen no. WY0801: (**a**) seed cones borne in opposite pairs, (arrow 1) two immature seed cones, scale bar = 5 mm; (**b**) & (**c**) two mature seed cones, (arrow 2) umbo of the scale, scale bar = 2 mm; (**d**) - (**f**) vegetative shoot, specimen no. WY0802: (**d**) vegetative shoot, scale bar = 5 mm; (**e**) branchlets, (arrow 3) the apex of facial leaf, (arrow 4) the apex of lateral leaf, scale bar = 2 mm; (**f**) branches, (arrow 5) the apex of facial leaf, scale bar = 2 mm

**Fig. 2:**
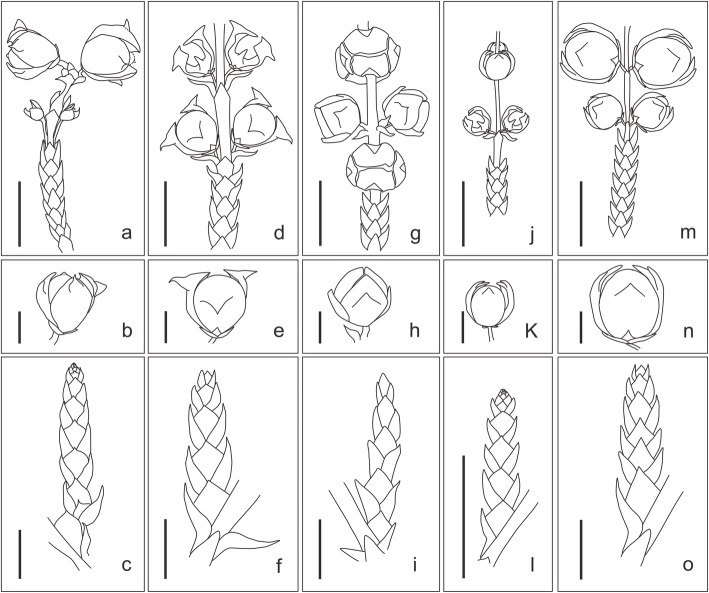
Line drawing of all 5 species of *Mesocyparis*. (**a**), (**b**) & (**c**) *M. sinica* (this paper); (**d**), (**e**) & (**f**) *M. borealis* (redrawn from [18]); (**g**), (**h**) & (**i**) *M. umbonata* (redrawn from [22]); (**j**), (**k**) & (**l**) *M. beringiana* (redrawn from [20, 22]); (**m**), (**n**) & (**o**) *M. rosanovii* (redrawn from [21]). (**a**), (**d**), (**g**), (**j**) & (**m**) fertile shoot of *Mesocyparis*, scale bar = 1 cm; (**b**), (**e**), (**h**), (**k**) & (**n**) seed cones of *Mesocyparis*, scale bar = 2 mm; (**c**), (**f**), (**i**), (**l**) & (**o**) branchlets in vegetative shoots of *Mesocyparis*, scale bar = 5 mm

**Diagnosis:** Seed cones ovate to orbicular, borne in opposite pairs (Fig. 1a and Fig.2a). Cone scales 4 in number, woody, obovate to ovate, decussate, approximately equal in size (Fig. 1b, c and Fig. 2b). Umbo near apex of scale, leaflike, erect, with apex tapered (Fig. 1b, c: arrow 2, 2a and 2b). Branches pinnate and opposite, forming flat frond-like sprays (Fig. 1d). Leaves on both vegetative and fertile shoots decussate, scaly and dimorphic with facial and lateral leaves (Fig. 1a, d, Fig. 2a and c).

**Holotype**: compression fertile shoot with two mature seed cones and two immature seed cones, Palaeobotanical Museum of China, Institute of Botany, Chinese Academy of Sciences (IBCAS), specimen no. WY0801 (Fig. 1)a, b and c.

**Etymology**: The specific name, *sinica*, refers to the fossil discovery in China.

**Fig. 3:**
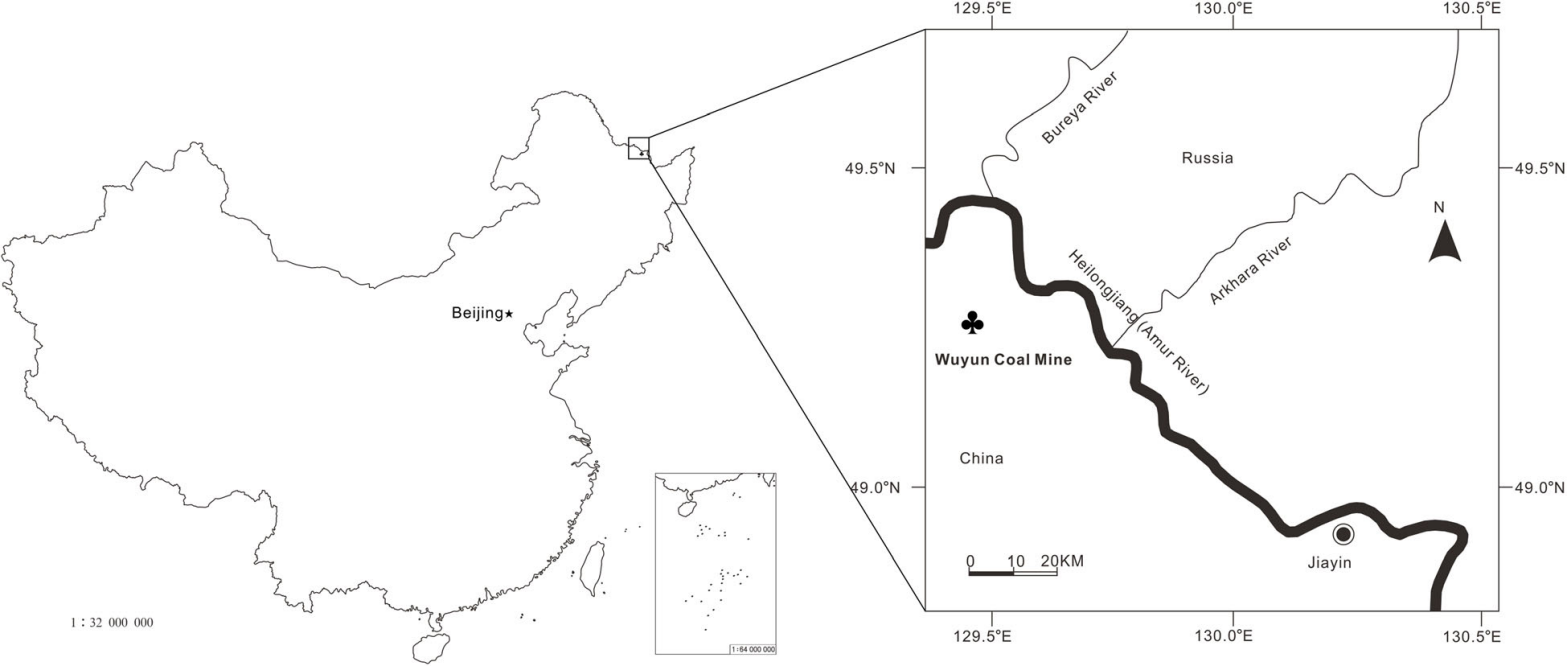
Locality of *Mesocyparis sinica*. Map showing the locality (♣) of *Mesocyparis sinica* in Jiayin, Heilongjiang, China. The base map of China Map is quoted from Geographic information systems of China website (http://bzdt.nasg.gov.cn/?ref=qqmap, map number: GS (2016)1570)

**Type locality**: Wuyun coalmine of Jiayin County, Heilongjiang Province, NE China (49° 14′ N, 129° 28′ E; Fig. 3).

**Horizon and age:** Wuyun Formation, Danian, Paleocene.

**Description:** Seed cones borne in opposite pairs, ovate, 4.4–5.2 mm long, 4.0–5.1 mm wide (Fig. 1a). Bracts 4 in number, woody, decussate, obovate to ovate, 3.7–4.3 mm long, 2.8–3.1 mm wide, and sizes of upper pair and basal pair nearly identical (Fig. 1b and c). Umbo leaflike, erect, at top of scale, with apex acuminate (Fig. 1b and c: arrow 2). Pair of immature seed cones arising from axis of fertile branch measure, 1.4–1.5 mm long, 0.9–1.3 mm wide (Fig. 1a: arrow 1). Immature cone scales peltate, 0.7–1.5 mm long, 0.8–0.9 mm wide, apex abrupt to blunt.

Fertile shoots flattened, leaves decussate and dimorphic, facial and lateral leaves morphologically different (Fig. 1a). Facial leaves triangular to rhombic, 0.9–2.7 mm long, 0.7–1.2 mm wide, thickened along midrib. Tips of leaves are acute to blunt, rising above basal part of subsequent leaves. Lateral leaves folded, falcate to triangular, 1.2–2.2 mm long, 0.4–0.8 mm wide, apex acute, rising above basal part of subsequent lateral leaves (Fig. 1a).

Branching pinnate, flattened, branchlets plagiotropic, borne in opposite pairs, forming flat frond–like sprays, about 3.2 cm long. Basal pair of facial leaves arising from the axils of branch lateral leaves. Branchlets on same side parallel, 1.9–2.8 mm apart (Fig. 1d). Scale-like leaves dimorphic, facial leaves adpressed, lateral leaves folded along midrib. There are some morphological differences between leaves of branches and branchlets (Fig. 1e and f). Facial leaves of branchlets ovate to rhombic, 1.1–2.1 mm long, 0.8–1.6 mm wide, adpressed, thickened along midrib, apex acuminate to acute, rising above basal part of subsequent leaves. Lateral leaves of branchlets folded, falcate, 0.9–2.4 mm long, 0.4–0.8 mm wide, apex acute; most of them overlap basal part of subsequent lateral leaves (Fig. 1e). Facial leaves of branches 1.8–3.8 mm long, 0.4–0.6 mm wide, apex acuminate to acute, thickened slightly, rising slightly above basal part of subsequent leaves. Lateral leaves of branches folded, falcate, 1.7–2.7 mm long, 0.4–0.5 mm wide, with apex acute, and basal part covered by earlier facial leaves (Fig. 1f).

### Comparison between *M. sinica* and other species of *Mesocyparis*

The newly-found fossils from Jiayin and other *Mesocyparis* records (Figs. 1 and 2) display significant differences (Fig. 2 and Table 1): The seed cones in the Jiayin fossil were erect, with an acuminate apex, while those of *M. borealis* from Saskatchewan, Canada were reflexed, with an acute apex [18]. Seed cones from Jiayin were borne in opposite pairs, with cone scales ovate to obovate, and umbo near the apex of the cone scale, while the leaves of fertile shoots were scaly and dimorphic. In contrast, the seed cones of *M. umbonata* from Alberta, Canada were decussate, with peltate cone scales bearing a central umbo, and leaves of fertile shoots monotypic [22]. The cone scales of the Jiayin specimens are nearly equal in size, while the upper pair of cone scales of *M. beringiana* from Koryak, Russia was larger than the basal pair, while the apex of the umbo was acute. At the same time, the seed cones and foliage of *M. beringiana* were smaller than those of the other three species and the Jiayin fossils [19, 20, 22]. Most cone scales of *M. rosanovii* from the Amur Region, Russia were arranged in decussate pairs, only a few being borne in opposite pairs. Moreover, the cone scales were peltate, with an acute umbo apex, and the leaves of the fertile shoots monotypic.

**Table 1:** Morphological comparison between fossil species of *Mesocyparis*

Taxa	*M. sinica*	*M. borealis*	*M. umbonata*	*M. beringiana*	*M. rosanovii*
Seed Cone					
Length (mm)	4.4–5.2	3.0–5.0	2.8–5.0	2.0–4.0	3.5–5.0
Width (mm)	4.0–5.1	3.0–5.0	1.9–4.0	2.0–4.0	3.0–4.0
Arrangement	opposite	opposite	decussate*	decussate*	most decussate, a few opposite*
Cone Scale					
shape	obovate to ovate	obovate	peltate*	ovate	peltate*
Length (mm)	3.7–4.3	3.0–5.0	2.3–4.0	no data	no data
Width (mm)	2.8–3.1	3.0	1.8–3.9	no data	no data
number	4	4	4	4	4
size of the two pairs	approximately equal in size	approximately equal in size	upper pair larger than lower*	upper pair larger than lower*	approximately equal in size
position of umbo on cone scale	near the apex	near the apex	in center*	near the apex	near the apex
Shape of umbo’ s tip	acuminate	acute*	acute*	acute*	acute*
Shape of umbo	erect	reflexed*	erect	erect	erect
Branch					
Arrangement	opposite, forming flat frond-like sprays	opposite, forming flat frond-like sprays	more than 1/4 alternate, others opposite*	opposite, forming flat frond-like sprays	most opposite, a few alternate*
Leaf					
Facia and lateral leaves of fertile shoots	dimorphic	dimorphic	monomorphic*	dimorphic	monomorphic*
Shootlet					
Length of facial leaves (mm)	1.1–2.1	2.0–3.0	0.8–2.0	0.5–0.6	1.5
Width of facial leaves (mm)	0.8–1.6	1.2–2.0	0.6–1.2	no data	1.0
Length of lateral leaves (mm)	0.9–2.4	2.0–3.0	0.5–2.0	no data	no data
Width of lateral leaves (mm)	0.4–0.8	0.5–1.0	0.1–0.4	no data	no data
facial leaves overlap	Present	Present	Present	Absent*	Present
lateral leaves overlap	Present	Present	Present	Absent*	Present
Shoot					
Length of facial leaves (mm)	1.8–3.8	3.5–6.0	1.4–4.0	2.0–3.0	1.5–3.0
Width of facial leaves (mm)	0.4–0.6	0.5–1.0	0.5–0.9	1.5–2.0	0.8–2.0
Length of lateral leaves (mm)	1.7–2.7	3.5–5.0	1.5–3.8	no data	1.5–3.0
Width of lateral leaves (mm)	0.4–0.5	0.5–1.0	0.2–0.6	no data	0.4–0.8
facial leaves overlapping	Present	no data	Absent*	no data	Absent*
facial leaves appressed	Present	Present	Present	no data	Present
lateral leaves overlap	Absent	Absent	Absent	no data	Absent
apices of lateral leaves free	Present	Present	Present	no data	Present
Location	Heilongjiang, China	Saskatchewan, Canada	Alberta, Canada	Koryak, Russia	Amur Region, Russia
Age	Early Paleocene	Paleocene	Masstrichtian	Masstrichtian	Early Paleocene
Reference	this paper	[18]	[22]	[19, 20, 22]	[21]

“*” indicating character differences between *Mesocyparis sinica* and other four *Mesocyparis* species

Here we provide a key to the fossil species of *Mesocyparis* based on cone characters as below: 
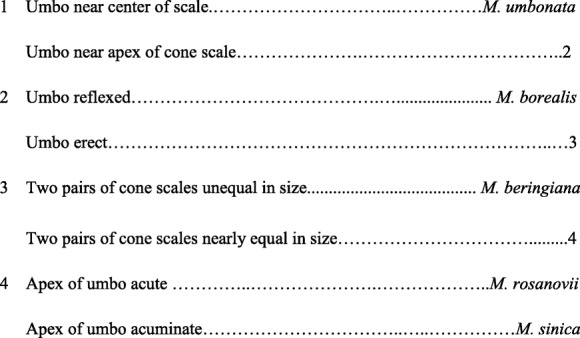


### Phylogenetic position of *Mesocyparis*

Here we treat the Wuyun fossils as a new species based on a detailed morphological comparison, which shows two or more significant morphological differences between the Wuyun fossils and other *Mesocyparis* species (Fig. 2, Table 1 and the Key in “Comparison between *M. sinica* and other species of *Mesocyparis*” section). The full fossil description and a morphological comparison with the other four species in *Mesocyparis* are provided in supplementary text of the electronic supplementary material.

**Fig. 4:**
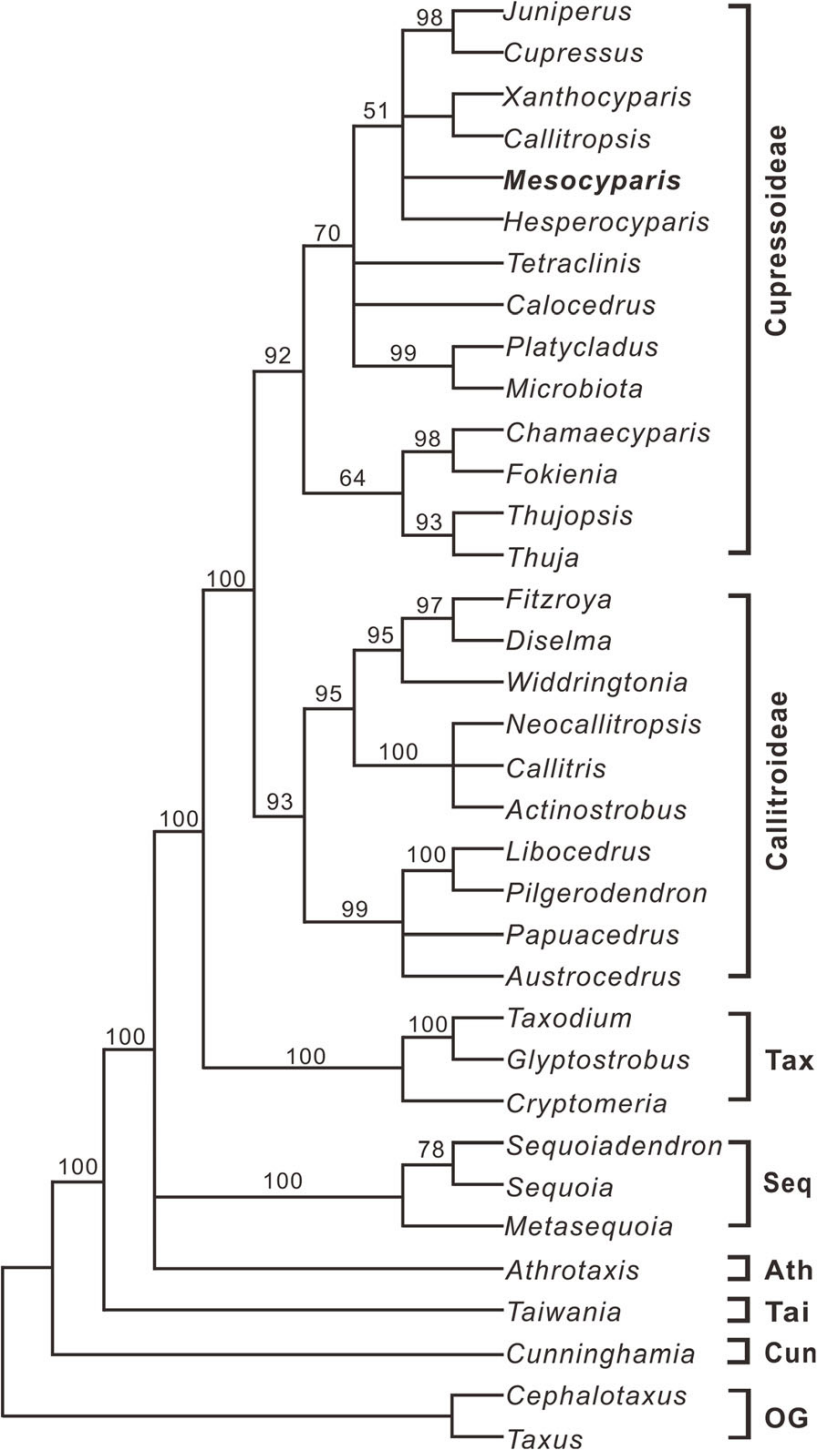
The strict consensus tree obtained after addition of *Mesocyparis* (in bold) to the result of the two trees [48, 49]. Numbers above the branches are bootstrap values (> 50%). Tax, Taxodioideae; Seq, Sequoioideae; Ath, Athrotaxidoideae; Tai, Taiwanioideae; Cun, Cunninghamioideae; Og, outgroups

**Fig. 5:**
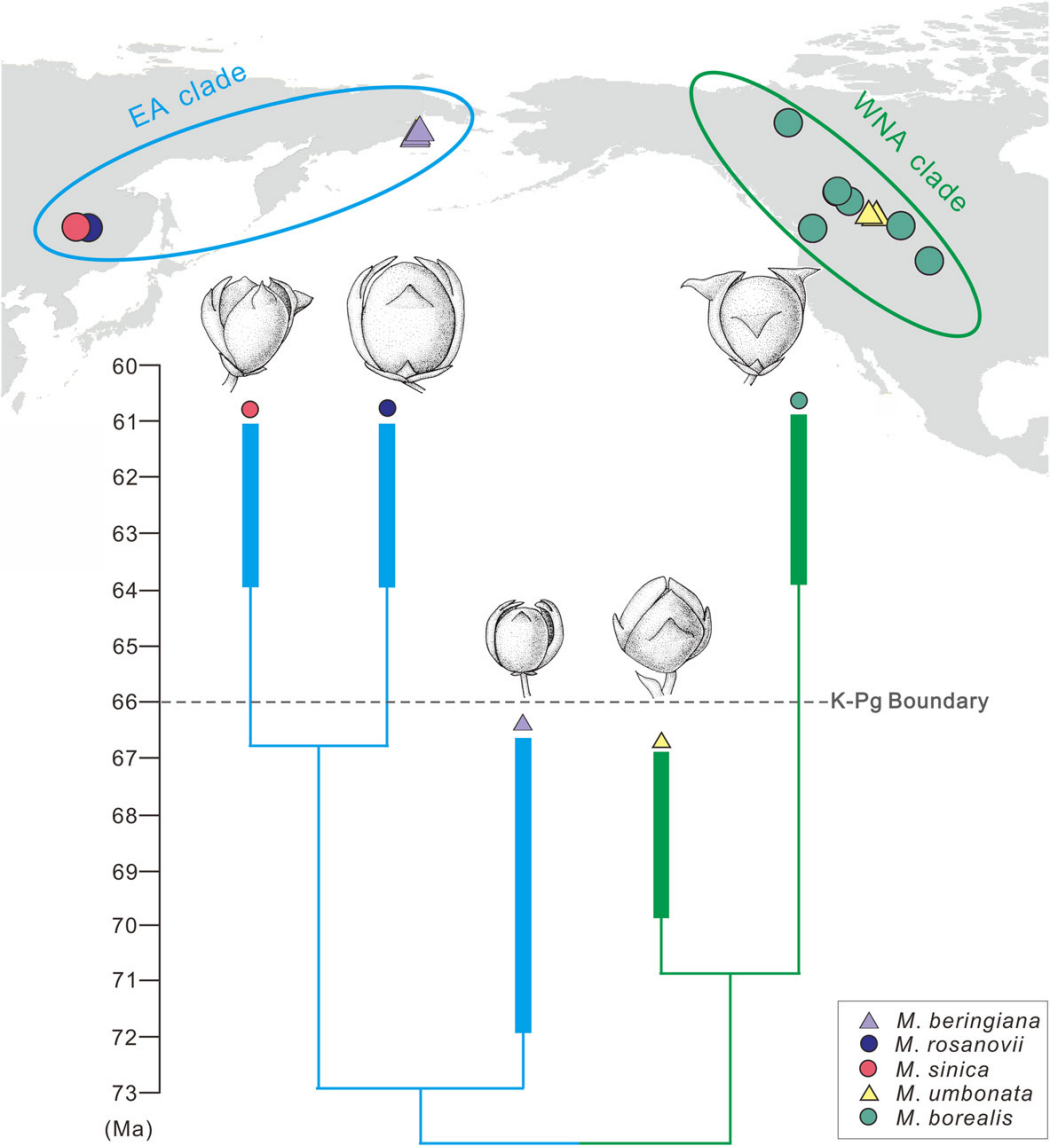
Time-scaled phylogeny, distribution and seed cone’s morphological changes of *Mesocyparis*. This figure shows obvious morphological changes of *Mesocyparis* in *ca.* Ten million years during the K-Pg transition. The cladogram is simplified from the strict consensus tree obtained from the phylogenetic analysis. The bars represent the dating of upper and lower limits of terminal taxon-bearing deposits, and a minimum branch length of 1 Ma based on the “minimum branch length” method used in [53]

Our family-wide phylogenetic analysis indicates that *Mesocyparis* is a member of the Cupressoideae and clusters with *Hesperocyparis*, *Callitropsis–Xanthocyparis*, and *Juniperus–Cupressus* (BS = 51%) (Fig. 4). *Mesocyparis* shares whorled cone scales equal (Additional file 1: Char. 30:1 and Table S1), and ovule shape obpyriform (Additional file 1: Char. 34:2 and Table S1), with these five genera. The phylogenetic tree of *Mesocyparis* is shown in Fig. 5, Additional file 2: Figure. S1 and Additional file 3: Table S2. In *Mesocyparis*, the two western North American species, *M. borealis* and *M. umbonata*, formed a clade (WNA clade, BS = 70%), and the three Asian species, *M. beringiana, M. rosanovii* and *M. sinica*, formed another (EA clade, BS = 65%). *Mesocyparis sinica* is sister to *M. rosanovii* (BS = 62%).

### Distributional changes during the K-Pg transition

**Fig. 6:**
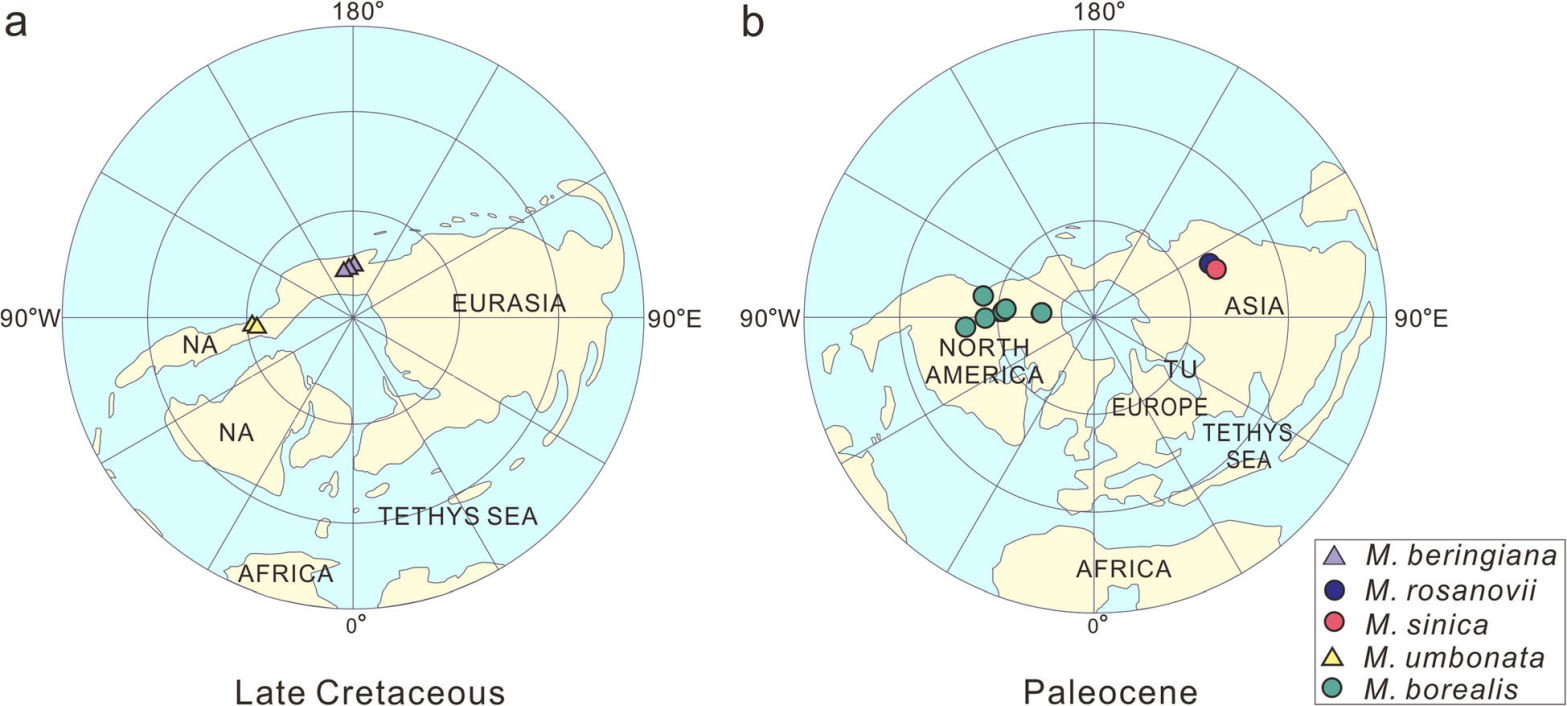
Paleogeographic maps of *Mesocyparis*. (**a**), Late Cretaceous; (**b**) Paleocene. NA = North America; TU = Turgai Straits. The data of paleolatitude and paleolongitude were converted from those of latitude and longitude of fossil sites using “PointTracker for Windows” software, and plotted on 2 individual paleogeographical maps on Projections of Lambert Equal-Area Azimuthal (North Pole) by using ArcGIS 3.2 software. The base maps of the late Cretaceous and the Paleocene were modified from Scotese [23]

Paleomaps [23] indicate that the landmasses of Eastern Asia and western North America were connected from the Late Cretaceous to the Paleocene. However, North America in the Late Cretaceous was divided by the Western Interior Seaway, while in the Paleocene western and eastern North America became contiguous due to the retreat of this seaway. Fossil records show *Mesocyparis* ranged from 59°N–76°N paleolatitude (Horseshoe Canyon, Alberta, Canada [18] and Kyoyak, Russia [18–20] during the Late Cretaceous (Fig. 6a) while in the Paleocene it ranged from 52 to 72°N paleolatitude (Amur, Russia [21] and Heilongjiang, China (this study) in Asia and Wyoming & Alaska, USA and Alberta, Canada [22] of western North America) (Fig. 6b). This *Mesocyparis* distribution (Fig. 6), combined with phylogenetic analysis (Fig. 5), reveals that the genus probably first occurred at high latitudes in the Northern Hemisphere and then divided into 2 clades —— the East Asian (EA) clade and the Western North American (WNA) clade.

## Discussion

### Morphological changes during the K-Pg transition

When plotting the scored 22 morphological characters against the species-level tree of *Mesocyparis*, we found some significant morphological changes in the genus from Maastrichtian to Paleocene: 1) seed cones became larger (average cone size increased about 130%, detail estimates in Methods), 2) the umbo shape on the scale changed from erect (Fig. 2h and k) to reflexed (Fig. 2e), and 3) the umbo position on the cone scales moved from the middle (Fig. 2h and k) to the apex (Fig. 2e).

**Fig. 7:**
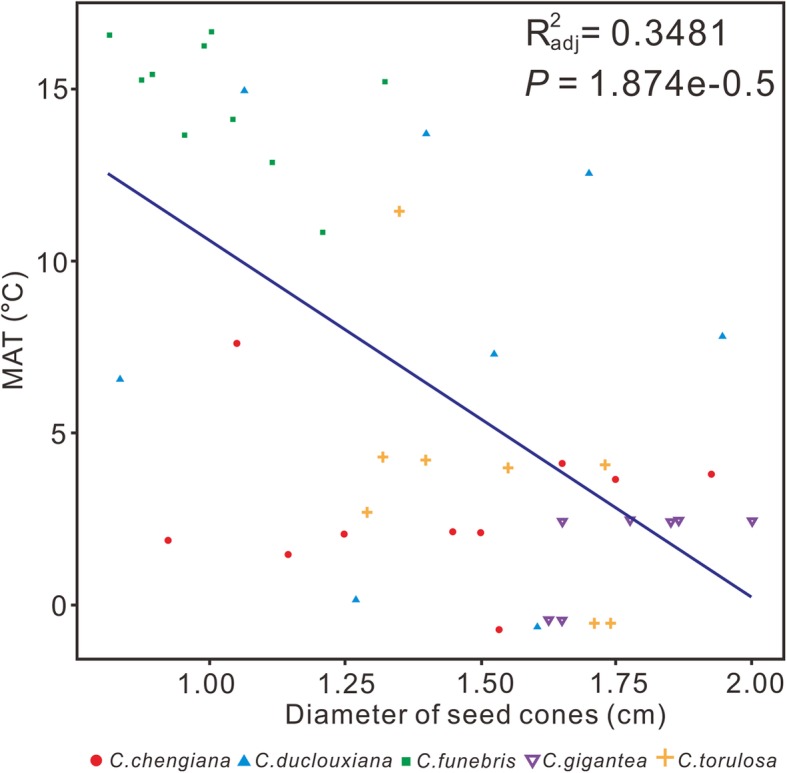
The relationship between the diameters of the *Cupressus* cones and mean annual temperature (MAT)

In general, the increasing size of the seed cone in *Mesocyparis* may be associated with the climate cooling from Maastrichtian to Paleocene across the K-Pg boundary. A negative correlation between the cone size and mean annual temperature (MAT) is apparent in modern *Cupressus*, one of the nearest living relatives of extinct *Mesocyparis* (Fig. 7 and Additional file 4: Table S3). In addition, it has been observed that fruits and seeds of angiosperms in North America and Europe increased during the K-Pg transition [24].

Specific morphological features can be linked to a specific biological function, and may allow us to speculate about the reason behind *Mesocyparis* seed cones more than doubling in size across the K-Pg transition. The increasing size of seed cones might augment their visibility and attractiveness for herbivorous and/or omnivorous animals, thereby enhancing seed dispersal, which could expand the plant distribution and increase their ability to survive temporal and spatial environmental change. Today some bird species (such as azure-winged magpie and light-vented bulbul) feed on intact seed cones and we envisage similar feeding strategies existed in early birds during the K-Pg transition. The increasing size of the seed cone might be an adaptive mechanism for seed dispersal (i.e., conspicuously larger seed cones were more attractive for those cone-eating/removal animals). An animal’s preference for larger seed cones might select and even amplify the increasing size of seed cones in *Mesocyparis*. Evidence has shown that the rapid radiation and diversification of birds [25, 26] and mammals [27] that, occurred during this period produced numerous potential vectors for seed dispersal of both gymnosperms and angiosperms.

### Cooling during the K-Pg transition drove *Mesocyparis* southwards

In general, the geographical distribution of plants at high latitudes is strongly constrained by the terrestrial thermal regime, particularly the mean coldest monthly temperature (MCMT) and/or the mean annual temperature (MAT) [28, 29]. Thus, the northern limit of a specific terrestrial plant taxon can reflect temperature changes with relatively high sensitivity [30].

**Fig. 8:**
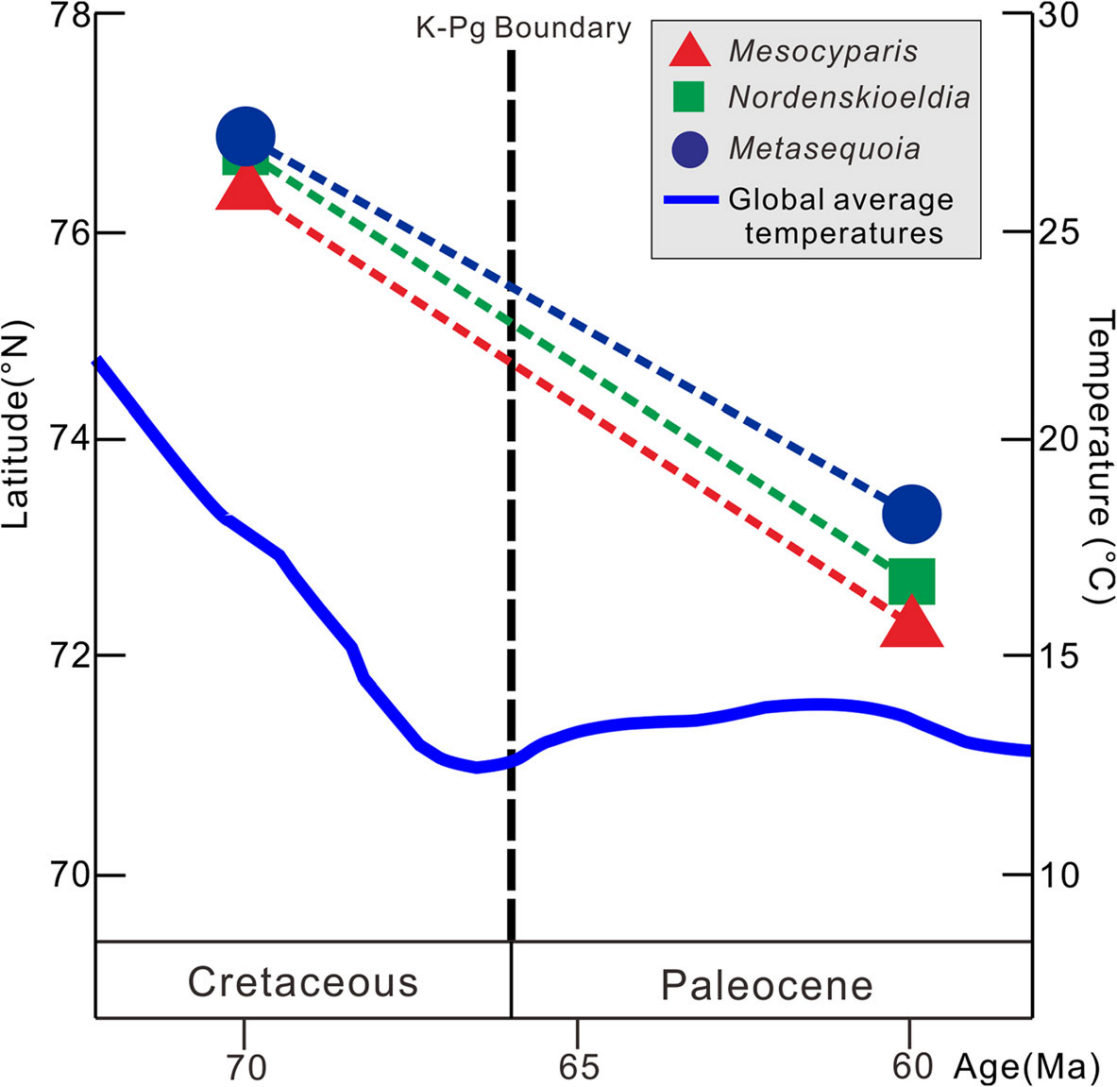
The paleolatitudinal distribution of *Mesocyparis*, *Metasequoia* and *Nordenskiodia*. This figure shows that the northern limits of these plant taxa migrated southward the cooling during the K-Pg transition. The depiction of global average temperature changes is modified from [31]

The cooling during the K-Pg transition (ca. 70–60 Ma, global average temperature fell by ca. 4 °C) [31] could have driven the northern limit of *Mesocyparis* south by ca. 4° N from the Maastrichtian to the Paleocene (Fig. 8 and Additional file 5: Table S4), which means that the northern limit of survival for the plant retreated southwards by ca. 444 km. Similar responses are found in *Metasequoia* (Cupressaceae) and *Nordenskioldia* (Trochodendraceae). *Metasequoia*’s northern limit moved south by ca. 4 ° (Fig. 8 and Additional file 6: Table S5), while *Nordenskioldia’s* moved almost 5 ° (Fig. 8 and Additional file 7: Table S6). These findings indicate that the southward migration of the northern limit of *Mesocyparis* as driven by the cooling during the K-Pg transition is by no means unique.

## Conclusions

We report a new fossil species, *Mesocyparis sinica* from the early Paleocene of Jiayin, Heilongjiang, northeastern China. By integrating lines of evidence from phylogeny and the comparative morphology of *Mesocyparis*, we found that during ca. 70–60 Ma, the size of the seed cone of *Mesocyparis* increased by ca. 130%, probably driven by the cooling during the K-Pg transition, which might be an effective adaptation for seed dispersal by animals. Meanwhile, our analyses indicate that the northern limits of this genus, as well as those of two other arboreal taxa *Metasequoia* (gymnosperm) and *Nordenskioldia* (angiosperm), have migrated ca. 4–5° southwards in paleolatitude during this interval, in association with the cooling. This is the first report to link plant evolutionary and ecological responses to the climate cooling during the K-Pg transition. This study not only enriches our understanding of how specific plant taxa responded to the climate changes during the K-Pg transition, but also provides a reversed case study for the scientific community to speculate about plant responses and distributional changes under global warming today.

## Methods

### Geological setting

The specimens of *Mesocyparis sinica* described here were collected from the upper part of the Wuyun Formation, at Wuyun coalmine (49° 14′ N, 129° 28′ E; Fig. 3) in Jiayin County, Heilongjiang Province, NE China. The enclosing sediments of the Jiayin fossil are grey sandy mudstone. Although some authors [32, 33] suggested it was of Maastrichtian (Late Cretaceous) age, the Wuyun Formation is now considered to belong to the Danian stage of the Early Paleocene based on palynotology [34–36], vegetation composition [37–40] and stratigraphic correlation [41–43]. In addition, Single zircon LA-ICP-MS dating shows the age of the lower part of the Wuyun Formation to be 64.1 ± 0.7 Ma [44, 45].

### Materials in this study

The fossil specimens of *Mesocyparis sinica* examined in this article are deposited in the National Museum of Plant History of China, Institute of Botany, Chinese Academy of Sciences: WY0801 (Figs. 1a, b and c), WY0802 (Figs. 1d, e and f), WY0803. These specimens were exposed and observed under a stereomicroscope, which identified by Dr. Yi-Ming Cui and Prof. Yu-Fei Wang. The characters were described following the terminology of [46]. Attempts were made to extract structure of seed cones under micro-CT and cuticles of leaf, but these efforts were unsuccessful.

Morphological and distributional information for the other four species of extinct *Mesocyparis* were collected from literature: *M. borealis* from [18], *M. umbonata* from [22], *M. beringiana* from [19, 20, 22], and *M. rosanovii* from [21].

The morphological and distributional information of extant *Cupressus* were measured and collected from specimens in the Herbarium of the Institute of Botany, Chinese Academy of Sciences, Beijing (PE) (Additional file 1: Table S1).

### Phylogenetic analysis

We first used the “backbone constraint” tree approach [47] to determine the phylogenetic position of *Mesocyparis* in the Cupressaceae *s.l.* The constrained tree is modified from the two trees of Mao et al. [48] and Yang et al. [49], in which significant conflicting nodes (BS ≥ 85%) between the two trees were collapsed. The morphological matrix was modified from Farjon [46] and included 53 characters (Additional file 1: Table S1). Following the family-wide analysis, we selected *Juniperus monticola*, *Xanthocyparis vietnamensis*, and *Hesperocyparis arizonica* as outgroups and built the species-level relationships of *Mesocyparis*. All five species of *Mesocyparis* were included. The 22 morphological characters were coded based on the literature [18–22] and our observations (Additional file 3: Table S2). Phylogenetic analysis for each matrix was conducted using maximum parsimony method in PAUP* version 4.0b10 [50]. Heuristic searches were performed with 1000 random sequence addition replicates, tree-bisection-reconnection branch swapping, MulTrees in effect, and steepest descent off. Bootstrapping was carried out with 1000 replicates, using a heuristic search strategy.

### Estimates of average cone size changes during the K-Pg transition

Here we use a volume formula of an ellipsoid to estimate the average cone size of each species in *Mesocyparis*, for the shapes of the seed cones in this genus are ovoid to ellipsoidal. Therefore, we set the seed cone length (*scl*) as the polar-radius of the ellipsoid and seed cone width (*scw*) as the two equatorial-radii. The estimated volume (*V*) of *Mesocyparis* is expressed by the following ellipsoid volume equation:

$$ V=\frac{4}{3}\uppi \times \frac{1}{2} scl\times \frac{1}{2} scw\times \frac{1}{2} scw=\frac{\pi }{6}\times scl\times {scw}^2 $$

where *scl* = median value of seed cone length, *scw* = median value of seed cone width. The *scl*s of five species in *Mesocyparis* are 3.9 mm (*M. umbonata*), 3 mm (*M. beringiana*), 4 mm (*M. borealis*), 4.25 mm (*M. rosanovii*) and 4.8 mm (*M. sinica*), while their *scw*s are 2.95 mm (*M. umbonata*), 3 mm (*M. beringiana*), 4 mm (*M. borealis*), 3.5 mm (*M. rosanovii*) and 4.55 mm (*M. sinica*). Based on the volume equation, the estimated volumes are 5.657 mm^3^ (*M. umbonata*), 4.5 mm^3^ (*M. beringiana*), 10.667 mm^3^ (*M. borealis*), 8.677 mm^3^ (*M. rosanovii*) and 16.562 mm^3^ (*M. sinica*). Thus, we can estimate that the average volumes of *Mesocyparis* in the Maastrichtian and the Paleocene were 5.079 mm^3^ and 11.969 mm^3^ respectively, so the increase in volume of *Mesocyparis* from the Maastrichtian to the Paleocene was about 136%.

### Correlational analysis of seed cone sizes of *Cupressus* and temperature

To explore the influence of climate change on the morphology of seed cones in *Mesocyparis*, we calculated the potential relationship between the seed cone sizes of extant *Cupressus* and temperatures in their habitats. We examined 45 samples of five *Cupressus* species (*C. chengiana* S. Y. Hu, *C. funebris* Endl., *C. duclouxiana* Hickel, *C. torulosa* D. Don and *C. gigantea* Cheng et L. K. Fu) naturally occurring in China from PE Herbarium, which have female cones and entire information on locality and elevation. The diameters of the female cones were measured to represent their seed cone sizes. Mean annual temperature (MAT) used the climate data set of New et al. [51] and then corrected for the altitude of the plant locality using a temperature lapse rate of 5 °C /km (Additional file 4: Table S3).

### Paleogeographic analysis

The coordinates of paleolatitude and paleolongitude were compiled from previous reports of *Mesocyparis* [18–22], *Metasequoia* (Cupressaceae, gymnosperm) [17] and *Nordenskioldia* (Trochodendraceae, angiosperm) [52] in the literature, and converted from today’s latitude and longitude to those inferred for the Cretaceous and Paleocene using “Point Tracker for windows” software, to plot the sites on paleogeographic maps representing two intervals of geologic time. The base maps with paleocoastlines were modified from paleogeographic maps of the late Cretaceous (ca. 94 Ma) and the Paleocene (ca. 60 Ma) from Scotese [23].

## Additional files

Additional file 1: **Table S1.** Morphological characters matrix including both extant and extinct species of the Cupressaceae *s.l. (DOCX 34 kb)*

Additional file 2: **Fig. S1.** The strict consensus tree of *Mesocyparis* inferred from the 22 morphological characters. (TIF 337 kb)

Additional file 3: **Table S2.** Morphological characters matrix of *Mesocyparis* and its outgroups. (DOCX 17 kb)

Additional file 4: **Table S3.** The seed cones sizes of *Cupressus*, their geographic information and mean annual temperature (MAT) of their habitats. (DOCX 19 kb)

Additional file 5: **Table S4.** Fossil localities of *Mesocyparis* and the estimates of their paleo-latitudes and palaeo-longitudes. (DOCX 15 kb)

Additional file 6: **Table S5.** Fossil localities of *Metasequoia* and the estimates of their paleo-latitudes and palaeo-longitudes. (DOCX 20 kb)

Additional file 7: **Table S6.** Fossil localities of *Nordenskioldia* and the estimates of their paleo-latitudes and paleo-longitudes. (DOCX 16 kb)

## Data Availability

All data generated or analysed during this study are included in this published article and its additional files.

## Abbreviations

BS: Bootstrap
*ca*.: Circa
EA: East Asian
IBCAS: Institute of Botany, Chinese Academy of Sciences
K-Pg: Cretaceous – Paleogene
Ma: Megaannus = Million years ago
MAT: Mean annual temperature
MCMT: Mean coldest monthly temperature
PE: Herbarium of the Institute of Botany, Chinese Academy of Sciences, Beijing
*s.l.*: Sensu lato
*scl*: Seed cone length
*scw*: Seed cone width
*V*: Volume
WNA: Western North American
