# Supplementary material for: Fossil evidence reveals how plants responded to cooling during the Cretaceous-Paleogene transition
Source: BMC Plant Biol. 2019 Sep 13;19:402. doi: 10.1186/s12870-019-1980-y (PMC6743113; doi:10.1186/s12870-019-1980-y)
Supplement: Supplementary file 1 — Table S1. Morphological characters matrix including both extant and extinct species of the Cupressaceae s.l. (DOCX 34 kb) [file 12870_2019_1980_MOESM1_ESM.docx]

**Additional file 1.** Morphological character state descriptions among species of the Cupressaceae *s.l.*

**Table S1.** Morphological characters matrix including both extant and extinct species of the Cupressaceae *s.l.*

| **Characters** | **1** | **2** | **3** | **4** | **5** | **6** | **7** | **8** | **9** | **10** | **11** | **12** | **13** | **14** | **15** | **16** | **17** | **18** |
| --- | --- | --- | --- | --- | --- | --- | --- | --- | --- | --- | --- | --- | --- | --- | --- | --- | --- | --- |
| *Actinostrobus pyramidalis* | 0 | 0 | 0 | 0 | 2 | 2 | 0 | 0 | 0 | 1 | 0 | 0 | 0 | 0 | 0 | 2 | 0 | 1 |
| *Athrotaxis laxifolia* | 1 | 0 | 0 | 0 | 1 | 2 | 0 | 0 | 2 | 1 | 0 | 0 | 0 | 1 | 0 | 4 | 0 | 1 |
| *Austrocedrus chilensis* | 1 | 1 | 0 | 1 | 0 | 1 | 1 | 0 | 0 | 1 | 0 | 2 | 0 | 1 | 0 | 2 | 1 | 1 |
| *Calocedrus decurrens* | 1 | 0 | 0 | 1 | 0 | 1 | 1 | 0 | 2 | 1 | 1 | 2 | 1 | 1 | 1 | 2 | 0 | 1 |
| *Chamaecyparis lawsoniana* | 1 | 0 | 0 | 1 | 0 | 1 | 1 | 0 | 2 | 1 | 1 | 2 | 1 | 1 | 0 | 12 | 0 | 1 |
| *Callitropsis nootkatensis* | 1 | 0 | 0 | 1 | 0 | 1 | 1 | 0 | 2 | 1 | 1 | 2 | 1 | 1 | 0 | 0 | 0 | 1 |
| *Cryptomeria japonica* | 1 | 0 | 0 | 0 | 1 | 12 | 0 | 0 | 2 | 1 | 0 | 1 | 0 | 1 | 1 | 4 | 0 | 01 |
| *Cunninghamia lanceolata* | 1 | 0 | 0 | 0 | 1 | 2 | 0 | 1 | 0 | 0 | 0 | 1 | 0 | 0 | 0 | 4 | 1 | 01 |
| *Diselma archeri* | 0 | 1 | 0 | 0 | 0 | 1 | 0 | 1 | 0 | 1 | 0 | 0 | 0 | 1 | 0 | 0 | 0 | 1 |
| *Fitzroya cupressoides* | 1 | 01 | 0 | 0 | 1 | 1 | 0 | 0 | 0 | 0 | 0 | 0 | 0 | 1 | 0 | 0 | 0 | 1 |
| *Fokienia hodginsii* | 1 | 0 | 0 | 1 | 0 | 1 | 1 | 1 | 2 | 1 | 1 | 0 | 0 | 1 | 0 | 2 | 1 | 1 |
| *Xanthocyparis vietnamensis* | 1 | 0 | 0 | 1 | 0 | 14 | 1 | 0 | 2 | 1 | 1 | 2 | 1 | 0 | 0 | 2 | 0 | 1 |
| *Glyptostrobus pensilis* | 1 | 0 | 01 0 | | 1 | 14 | 0 | 0 | 2 | ? | 0 | 1 | 0 | 1 | 1 | 2 | 0 | 1 |
| *Metasequoia glyptostroboides* | 1 | 0 | 1 | 1 | 0 | 0 | 0 | 1 | 0 | 1 | 1 | 1 | 0 | 1 | 0 | 2 | 1 | 1 |
| *Microbiota decussata* | 0 | 0 | 0 | 1 | 0 | 1 | 1 | 0 | 2 | 1 | 1 | 2 | 1 | 1 | ? | 0 | 0 | 1 |
| *Neocallitropsis pancherii* | 01 | 0 | 0 | 0 | 2 | 2 | 0 | 0 | 0 | 1 | 0 | 0 | 0 | 0 | 0 | 2 | 0 | 1 |
| *Papuacedrus papuana* | 1 | 0 | 0 | 1 | 0 | 1 | 1 | 0 | 0 | 1 | 1 | 2 | 0 | 1 | 0 | 1 | 2 | 1 |
| *Pilgerofendron uviferum* | 1 | 01 | 0 | 0 | 0 | 1 | 0 | 2 | 0 | 0 | 0 | 0 | 0 | 1 | ? | 1 | 2 | 1 |
| *Platycladus orientalis* | 1 | 0 | 0 | 01 | 0 | 1 | 1 | 0 | 0 | 1 | 1 | 1 | 1 | 1 | 0 | 1 | 0 | 1 |
| *Sequoia semprevirens* | 1 | 0 | 0 | 01 | 1 | 01 | 0 | 0 | 0 | 0 | 0 | 1 | 0 | 1 | 1 | 0 | 0 | 1 |
| *Sequoiadendron giganteum* | 1 | 0 | 0 | 0 | 1 | 1 | 0 | 0 | 0 | 0 | 0 | 1 | 0 | 1 | 1 | 0 | 0 | 1 |
| *Taiwania cryptomerioides* | 1 | 0 | 0 | 0 | 1 | 1 | 0 | 0 | 0 | 1 | 0 | 1 | 0 | 1 | 0 | 4 | 0 | 1 |
| *Taxodium distichum* | 1 | 0 | 1 | 01 | 1 | 02 | 0 | 0 | 2 | ? | 0 | 1 | 0 | 1 | 1 | 2 | 0 | 1 |
| *Tetraclinis articulata* | 01 | 0 | 0 | 0 | 0 | 1 | 1 | 2 | 2 | 1 | 1 | 2 | 1 | 0 | 1 | 2 | 2 | 1 |
| *Thujopsis dolobrata* | 01 | 0 | 0 | 1 | 1 | 1 | 1 | 02 | 0 | 1 | 1 | 0 | 0 | 1 | 0 | 0 | 0 | 1 |
| *Widdringtonia nodiflora* | 01 | 0 | 0 | 0 | 01 | 1 | 0 | 0 | 0 | 1 | 0 | 2 | 0 | 0 | 1 | 4 | 0 | 01 |
| *Hesperocyparis arizonica* | 01 | 0 | 0 | 01? | 0 | 1 | 1 | 0 | 2 | 1 | ? | ? | 1 | 1 | 1 | 2 | 0 | 1 |
| *Cupressus atlantica* | 1 | 0 | 0 | 0 | 0 | 1 | 0 | 0 | 2 | 1 | ? | ? | 1 | 0 | 0 | 0 | 0 | 1 |
| *Juniperus monticola* | 01 | 1 | 0 | 01 | 0 | 1 | 0 | 0 | 0 | 1 | ? | ? | ? | 0 | ? | ? | ? | 1 |
| *Thuja occidentalis* | 1 | 0 | 0 | 1 | 0 | 1 | 1 | 02 | 2 | 1 | 1 | 1 | 1 | 1 | 0 | 2 | 0 | 1 |
| *Callitris rhomboidea* | 01 | 0 | 0 | 0 | 2 | 4 | 0 | 2 | 0 | 1 | ? | ? | 0 | 0 | 0 | 2 | 0 | 1 |
| *Libocedrus bidwillii* | 01 | 0 | 0 | 1 | 0 | 1 | 1 | 0 | 2 | 1 | ? | ? | ? | 1 | 0 | 2 | ? | 1 |
| *Cephalotaxus sinensis* | 01 | 1 | 0 | 0 | 0 | 4 | 0 | 2 | 0 | ? | ? | ? | 0 | 1 | 0 | ? | ? | 0 |
| *Taxus cuspidata* | 1 | 0 | 0 | 0 | 1 | 4 | 0 | 1 | 0 | ? | 1 | ? | 0 | 1 | 0 | ? | ? | 0 |
| *Mesocyparis* | 1 | ? | 0 | 1 | 0 | 1 | 1 | 0 | 02 | 1 | 1 | 02 | 1 | 1 | ? | 0 | ? | 1 |

**Table S2. (*Cont.*)**

| **Characters** | **19** | **20** | **21** | **22** | **23** | **24** | **25** | **26** | **27** | **28** | **29** | **30** | **31** | **32** | **33** | **34** | **35** | **36** |
| --- | --- | --- | --- | --- | --- | --- | --- | --- | --- | --- | --- | --- | --- | --- | --- | --- | --- | --- |
| *Actinostrobus pyramidalis* | 0 | 2 | 1 | 1 | 0 | 1 | 0 | 2 | 1 | 2 | 1 | 1 | 1 | 0 | 1 | 1 | 1 | 0 |
| *Athrotaxis laxifolia* | 0 | 2 | 0 | 1 | 0 | 0 | 1 | 1 | 3 | 1 | 0 | - | 0 | 2 | 0 | 0 | 0 | 0 |
| *Austrocedrus chilensis* | 0 | 0 | 1 | 1 | 0 | 0 | 1 | 0 | 1 | 0 | 0 | 1 | 1 | 0 | 2 | 1 | 0 | 1 |
| *Calocedrus decurrens* | 0 | 0 | 1 | 1 | 0 | 0 | 1 | 0 | 0 | 0 | 0 | 1 | 0 | 0 | 1 | 3 | 0 | 0 |
| *Chamaecyparis lawsoniana* | 0 | 0 | 1 | 1 | 0 | 0 | 1 | 0 | 2 | 0 | 0 | 1 | 0 | 0 | 1 | 2 | 0 | 1 |
| *Callitropsis nootkatensis* | 0 | 0 | 0 | 1 | 0 | 0 | 1 | 0 | 0 | 0 | 0 | 1 | 1 | 0 | 1 | 2 | 0 | 1 |
| *Cryptomeria japonica* | 0 | 1 | 2 | 1 | 1 | 0 | 1 | 1 | 3 | 1 | 0 | - | 0 | 0 | 0 | 0 | 1 | 1 |
| *Cunninghamia lanceolata* | 1 | 1 | 1 | 1 | 1 | 0 | 1 | 1 | 3 | 1 | 0 | - | 0 | 2 | 0 | 2 | 0 | 0 |
| *Diselma archeri* | 0 | 0 | 0 | 1 | 0 | 1 | 1 | 0 | 1 | 0 | 0 | 1 | 1 | 0 | 1 | 1 | 0 | 0 |
| *Fitzroya cupressoides* | 0 | 2 | 2 | 1 | 0 | 0 | 1 | 2 | 2 | 0 | 0 | 1 | 1 | 0 | 1 | 2 | ? | 0 |
| *Fokienia hodginsii* | 0 | 0 | 1 | 1 | 0 | 0 | 1 | 0 | 2 | 0 | 0 | 1 | 0 | 0 | 1 | 2 | ? | 0 |
| *Xanthocyparis vietnamensis* | 0 | 0 | 1 | 1 | 0 | 01 | 1 | 0 | 0 | 0 | 0 | 1 | 01 | 0 | 1 | 2 | 0 | 1 |
| *Glyptostrobus pensilis* | 0 | 1 | 2 | 1 | 1 | 0 | 1 | 1 | 3 | 1 | 0 | - | 0 | 0 | 1 | 1 | 1 | 1 |
| *Metasequoia glyptostroboides* | 0 | 1 | 0 | 1 | 1 | 0 | 1 | 0 | 3 | 1 | 0 | 1 | 0 | 2 | 0 | 0 | 1 | 0 |
| *Microbiota decussata* | 0 | 0 | 0 | 1 | 0 | 0 | 1 | 0 | 1 | 0 | 0 | 1 | 0 | 0 | 1 | 3 | - | 0 |
| *Neocallitropsis pancherii* | 0 | 2 | 3 | 1 | 0 | 1 | 1 | 2 | 2 | 0 | 1 | 1 | 1 | 0 | ? | 1 | ? | 1 |
| *Papuacedrus papuana* | 0 | 02 | 2 | 1 | 0 | 0 | 1 | 0 | 1 | 0 | 0 | 1 | 1 | 0 | 2 | 1 | 0 | 1 |
| *Pilgerofendron uviferum* | 0 | 0 | 2 | 1 | 0 | 0 | 1 | 0 | 1 | 2 | 0 | 1 | 1 | 0 | 2 | 1 | 0 | 1 |
| *Platycladus orientalis* | 0 | 0 | 2 | 1 | 0 | 0 | 1 | 0 | 1 | 0 | 0 | 1 | 0 | 0 | 1 | 3 | 0 | 0 |
| *Sequoia semprevirens* | 0 | 1 | 0 | 1 | 1 | 0 | 1 | 1 | 3 | 1 | 0 | - | 0 | 2 | 0 | 0 | 1 | 0 |
| *Sequoiadendron giganteum* | 0 | 1 | 0 | 1 | 1 | 0 | 1 | 1 | 3 | 1 | 0 | - | 0 | 2 | 0 | 0 | 1 | 0 |
| *Taiwania cryptomerioides* | 1 | 1 | 1 | 1 | 1 | 0 | 1 | 1 | 3 | 1 | 0 | - | 0 | 2 | 0 | 0 | 0 | 0 |
| *Taxodium distichum* | 0 | 2 | 2 | 1 | 1 | 1 | 0 | 1 | 3 | 1 | 0 | - | 0 | 0 | 1 | 0 | 1 | 1 |
| *Tetraclinis articulata* | 0 | 1 | 0 | 1 | 0 | 0 | 01 | 0 | 1 | 0 | 1 | 1 | 0 | 0 | 1 | 2 | 0 | 0 |
| *Thujopsis dolobrata* | 0 | 1 | 1 | 1 | 0 | 0 | 1 | 0 | 2 | 0 | 0 | 1 | 0 | 0 | 1 | 3 | 1 | 0 |
| *Widdringtonia nodiflora* | 0 | 1 | 2 | 1 | 0 | 1 | 0 | 0 | 0 | 0 | 1 | 1 | 1 | 0 | 1 | 1 | 1 | 0 |
| *Hesperocyparis arizonica* | 0 | 0 | 12 | 1 | ? | 0? | 0 | 0 | 12 | 0 | 0 | 1 | 0 | 0 | 1 | 2 | ? | ? |
| *Cupressus atlantica* | 0 | 0 | 12 | 1 | ? | ? | 1 | 0 | 3 | 0 | 0 | 1 | 0 | 0 | 1 | 2 | ? | ? |
| *Juniperus monticola* | 0 | 0 | 12 | 1 | 0 | 0 | 1 | 0 | 0 | 0 | 0 | 1 | 0 | 0 | 1 | 2 | ? | ? |
| *Thuja occidentalis* | 0 | 0 | 12 | 1 | ? | 0 | 1 | 0 | 12 | 0 | 0 | 1 | 0 | 0 | 1 | 3 | ? | ? |
| *Callitris rhomboidea* | 1 | 2 | 12 | 1 | ? | 1 | 01 | 2 | 0 | 0 | 0 | 1 | 1 | 0 | 1 | 1 | 1 | 1 |
| *Libocedrus bidwillii* | 0 | 0 | 2 | 1 | ? | 0 | 1 | 0 | 1 | 0 | 0 | 1 | 1 | 0 | 2 | 1 | ? | ? |
| *Cephalotaxus sinensis* | 4 | ? | 1 | ? | ? | 0 | 01 | 0 | ? | ? | ? | ? | 0 | 0 | ? | ? | ? | ? |
| *Taxus cuspidata* | 2 | ? | 23 | ? | ? | 0 | 01 | 0 | ? | ? | ? | ? | 0 | 0 | 1 | ? | ? | ? |
| *Mesocyparis* | 5 | 0 | 012 | 01 | ? | 1 | 1 | 0 | 1 | 0 | 01 | 1 | 1 | 0 | 1 | 2 | ? | ? |

**Table S2. (*Cont.*)**

| **Characters** | **37** | **38** | **39** | **40** | **41** | **42** | **43** | **44** | **45** | **46** | **47** | **48** | **49** | **50** | **51** | **52** | **53** |
| --- | --- | --- | --- | --- | --- | --- | --- | --- | --- | --- | --- | --- | --- | --- | --- | --- | --- |
| *Actinostrobus pyramidalis* | 2 | 1 | 1 | 0 | 2 | 2 | 1 | 1 | 0 | 0 | 2 | 1 | 2 | 1 | 0 | 0 | 2 |
| *Athrotaxis laxifolia* | 0 | 1 | 1 | 0 | 1 | 2 | 1 | 1 | 0 | 0 | 2 | 1 | 1 | 1 | 0 | 0 | 0 |
| *Austrocedrus chilensis* | 1 | 1 | 1 | 0 | 2 | 2 | 3 | 2 | 0 | 0 | 1 | 1 | 1 | 0 | 1 | 1 | 0 |
| *Calocedrus decurrens* | 1 | 1 | 1 | 1 | 2 | 2 | 1 | 1 | 0 | 0 | 2 | 1 | 1 | 0 | 1 | 1 | 1 |
| *Chamaecyparis lawsoniana* | 0 | 1 | 1 | 1 | 1 | 2 | 1 | 1 | 0 | 0 | 2 | 1 | 1 | 1 | 0 | 0 | 0 |
| *Callitropsis nootkatensis* | 1 | 1 | 1 | 0 | 2 | 2 | 1 | 1 | 0 | 1 | 2 | 1 | 1 | 1 | 0 | 1 | 1 |
| *Cryptomeria japonica* | 1 | 1 | 1 | 0 | 1 | 1 | 2 | 0 | 0 | 0 | 2 | 1 | 1 | 1 | 1 | 0 | 0 |
| *Cunninghamia lanceolata* | 0 | 0 | 1 | 0 | 0 | 1 | 0 | 0 | 1 | 0 | 2 | 1 | 1 | 1 | 0 | 0 | 0 |
| *Diselma archeri* | 0 | 0 | 1 | 0 | 2 | 2 | 1 | 1 | 0 | 0 | 1 | 1 | 2 | 1 | 0 | 1 | 0 |
| *Fitzroya cupressoides* | 2 | 1 | 1 | 0 | 2 | 2 | 1 | 1 | 0 | 0 | 1 | 1 | 2 | 1 | 0 | 1 | 0 |
| *Fokienia hodginsii* | 1 | 1 | 1 | 1 | 1 | 2 | 1 | 1 | 0 | 0 | 2 | 1 | 1 | 1 | 1 | 0 | 0 |
| *Xanthocyparis vietnamensis* | 1 | 1 | 1 | 0 | 2 | 2 | 1 | 1 | 0 | 1 | 1 | 1 | 1 | 1 | 0 | 1 | 1 |
| *Glyptostrobus pensilis* | 1 | 1 | 1 | 0 | 1 | 2 | 2 | 0 | 0 | 0 | 2 | 1 | 0 | 2 | 1 | 0 | 3 |
| *Metasequoia glyptostroboides* | 0 | 1 | 1 | 0 | 1 | 2 | 1 | 1 | 0 | 0 | 2 | 1 | 1 | 1 | 0 | 0 | 3 |
| *Microbiota decussata* | 1 | 1 | 1 | 0 | 2 | 2 | 1 | 1 | 0 | 0 | 0 | 0 | - | - | - | 1 | 1 |
| *Neocallitropsis pancherii* | 2 | 1 | 1 | 0 | 2 | 2 | 1 | 1 | 0 | 0 | 1 | 1 | 2 | 1 | 0 | 0 | 0 |
| *Papuacedrus papuana* | 3 | 1 | 1 | 0 | 2 | 2 | 3 | 2 | 0 | 0 | 1 | 1 | 1 | 1 | 1 | 0 | 0 |
| *Pilgerofendron uviferum* | 3 | 1 | 1 | 0 | 2 | 2 | 3 | 2 | 0 | 0 | 1 | 1 | 1 | 1 | 1 | 0 | 0 |
| *Platycladus orientalis* | 1 | 1 | 1 | 0 | 2 | 2 | 1 | 1 | 0 | 0 | 3 | 0 | - | - | - | 1 | 2 |
| *Sequoia semprevirens* | 0 | 1 | 1 | 0 | 1 | 2 | 1 | 1 | 0 | 0 | 2 | 1 | 1 | 1 | 0 | 0 | 3 |
| *Sequoiadendron giganteum* | 0 | 1 | 1 | 0 | 1 | 2 | 1 | 1 | 0 | 1 | 2 | 1 | 1 | 1 | 1 | 0 | 0 |
| *Taiwania cryptomerioides* | 0 | 0 | 1 | 0 | 0 | 2 | 0 | 0 | 1 | 0 | 2 | 1 | 1 | 1 | 0 | 0 | 0 |
| *Taxodium distichum* | 1 | 1 | 1 | 0 | 1 | 2 | 2 | 0 | 0 | 0 | 2 | 1 | 2 | 1 | 1 | 0 | 3 |
| *Tetraclinis articulata* | 1 | 1 | 1 | 0 | 2 | 2 | 1 | 1 | 0 | 0 | 2 | 1 | 1 | 1 | 0 | 0 | 2 |
| *Thujopsis dolobrata* | 1 | 1 | 1 | 1 | 1 | 2 | 1 | 1 | 0 | 1 | 2 | 1 | 1 | 1 | 0 | 0 | 0 |
| *Widdringtonia nodiflora* | 0 | 1 | 1 | 0 | 2 | 2 | 1 | 1 | 0 | 0 | 2 | 1 | 1 | 1 | 1 | 0 | 1 |
| *Hesperocyparis arizonica* | ? | ? | 1 | 0 | 1 | ? | ? | ? | 0 | 1 | 4 | 1 | 1 | 1 | ? | ? | ? |
| *Cupressus atlantica* | ? | ? | 1 | 0 | 1 | ? | ? | ? | 0 | 1 | 3 | 1 | 1 | 1 | 0 | ? | ? |
| *Juniperus monticola* | ? | ? | 0 | 2 | 1 | 2 | 1 | 1 | 2 | 1 | 23 | 0 | ? | ? | ? | 1 | ? |
| *Thuja occidentalis* | ? | ? | 1 | 1 | 1 | 2 | ? | ? | 0 | 0 | 34 | 1 | 1 | 1 | 0 | ? | ? |
| *Callitris rhomboidea* | ? | ? | 1 | 0 | 2 | 2 | 1 | 1 | 0 | 0 | 23 | 1 | 12 | 1 | 0 | ? | ? |
| *Libocedrus bidwillii* | ? | ? | 1 | 0 | 2 | ? | ? | ? | 0 | ? | 2 | 1 | 1 | 1 | 1 | ? | ? |
| *Cephalotaxus sinensis* | ? | ? | 1 | 0 | 1 | ? | ? | ? | 0 | 1 | 1 | ? | ? | ? | ? | ? | ? |
| *Taxus cuspidata* | ? | ? | ? | ? | ? | ? | ? | ? | ? | 1 | 0 | ? | ? | ? | ? | ? | ? |
| *Mesocyparis* | ? | 1 | 1 | 1 | 1 | ? | ? | ? | 0 | ? | 123 | 1 | 1 | 1 | 01 | 1 | ? |

"?" indicates missing; "-" indicates inapplicable.

**Note: Morphological characters and character states of Table S2**

Morphological data are modified from Farjon [46].

1 Life form: shrub (0); tree (1)

2 Breeding system: monoecious (0); dioecious (1)

3 Foliage abscission: evergreen (0); deciduous (1)

4 Plagiotropic foliage: absent (0); present (1)

5 Phyllotaxis of mature leaves: decussate (0); spiral (1); whorled (2)

6 Leaf shape on ultimate branchlet: acicular (0); scale-like (1); lanceolate (2); subulate (3); linear (4)

7 Dimorphism in foliar leaves: absent (0); present (1)

8 Stomata position mature leaves: amphistomatic (0); hypostomatic (1); epistomatic (2)

9 Orientation of stomata: parallel to midrib (0); perpendicular to midrib (1); irregular (2)

10 Florin ring: absent (0); present (1)

11 Papillae on epidermis of stomata: absent (0); present (1)

12 Cycles of stomatal subsidiary cells: monocyclic (0); amphicyclic (1); both (2)

13 Leaf gland: absent (0); present (1)

14 Leaf margin: denticulate (0); entire (1)

15 Cotyledons: two (0); three or more (1)

16 Initia phyllotaxis on seedling: decussate (0); 'whorls-3' (1); 'whorls-4' (2); 'whorls-5' (3); spiral (4)

17 Stomata on primary leaves: amphistomatic (0); hypostomatic (1); epistomatic (2)

18 Pollen cone position: lateral (0); terminal (1)

19 Pollen cone aggregation (max. number): 1 (0); 2 (1); 3 (2); 4 (3); more than 4 (5)

20 Microsporophylls phyllotaxis: decussate (0); spiral (1); whorled (2)

21 Pollen sacs: two (0); "few (2-3)" (1); "several (4-5)" (2); "many (6-10)" (3)

22 Germination pore: absent (0); present (1)

23 Papillate germination pore: absent (0); present (1)

24 Persistence of ovuliferous cones: caducous (0); persistent (1)

25 Cone aggregation: clusters (0); solitary (1)

26 Bract scale phyllotaxis: decussate (0); spiral (1); whorled (2)

27 Number of ovuliferous cone scales: three or six (0); four or eight (1); nine to 16 (2); more than 17 (3)

28 Sterile cone scales: fewer than 4 (0); more than 4 proximal + distal (1); more than 4 proximal (2)

29 Equal length of mature cone scales: absent (0); present (1)

30 Whorled cone scales: unequal (0); equal (1)

31 Columella: absent (0); present (1)

32 Ovule orientation: erect (0); primarily inverted (1); secondarily inverted (2)

33 Ovule position: on bract (0); axillary to bract (1); on apex of cone axis (2); on ovuliferous scale (3)

34 Ovule shape: globose (0); cylindrical (1); obpyriform (2); ampulliform (3)

35 Ovule development: synchronic (0); asynchronic (1)

36 Micropyle: synchronic (0); asynchronic (1)

37 Mature micropyle: not lobulate (0); bilobulate (1); trilobulate (2); multilobulate (3)

38 Papillae on cone scale: absent (0); present (1)

39 Cone dehiscence: closed (0); open (1)

40 Mature cone scale: free (0); apically connate (1); all fused (2)

41 Mature cone scale: foliate (0); peltate (1); valvate (2)

42 Ovuliferous scale: initiating before ovule formation (0); initiating simultaneously (1); initiating after ovule formation (2)

43 Cone scale dominance: bract only (0); intercalary growth (1); including lobate protuberances (2); including ligulate protuberance (3); ovuliferous scale (4)

44 Cone scale fused: with lobes (0); with intercalary growth (1); with ligulate structure (2); with bract (3); free (4)

45 Mature cone: lignified (0); coriaceous (1); fleshy (2)

46 Seed maturation: in the first year (0); in the second year or later (1)

47 Seed number per cone scale: one (0); one or two (1); two to four (2); five to ten (3); more than ten (4)

48 Seed wings: absent (0); present (1)

49 Wing number: one (0); two (1); three (2)

50 Position of seed wing: distal (0); lateral (1); proximal (2)

51 Wing shape: symmetric (0); asymmetric (1)

52 Pitting of tangential walls in ray parenchyma: single large (0); several small (1)

53 Pits in transfusion tracheids: circular bordered (0); barred trabeculate (1); large irregular (2); with narrow borders (3)
