# Supplementary figures and images for: Fossil evidence reveals how plants responded to cooling during the Cretaceous-Paleogene transition
Source: BMC Plant Biol. 2019 Sep 13;19:402. doi: 10.1186/s12870-019-1980-y (PMC6743113; doi:10.1186/s12870-019-1980-y)

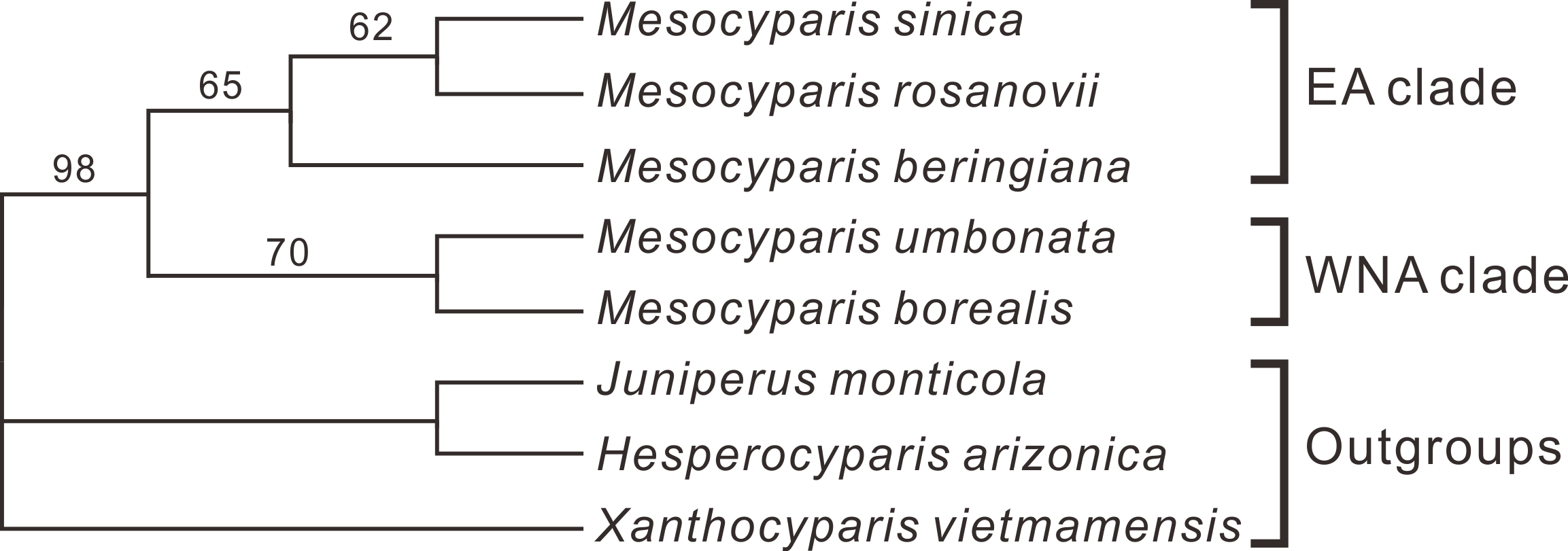

Supplement: Supplementary file 2 — Fig. S1. The strict consensus tree of Mesocyparis inferred from the 22 morphological characters. (TIF 337 kb) [file 12870_2019_1980_MOESM2_ESM.tif]
