# Supplementary material for: Fossil evidence reveals how plants responded to cooling during the Cretaceous-Paleogene transition
Source: BMC Plant Biol. 2019 Sep 13;19:402. doi: 10.1186/s12870-019-1980-y (PMC6743113; doi:10.1186/s12870-019-1980-y)
Supplement: Supplementary file 3 — Table S2. Morphological characters matrix of Mesocyparis and its outgroups. (DOCX 17 kb) [file 12870_2019_1980_MOESM3_ESM.docx]

**Additional file 3.** Morphological character state descriptions of species in *Mesovyparis*

**Table S2.** Morphological characters matrix of *Mesocyparis* and its outgroups

| **Taxa** | **1** | **2** | **3** | **4** | **5** | **6** | **7** | **8** | **9** | **10** | **11** | **12** | **13** | **14** | **15** | **16** | **17** | **18** | **19** | **20** | **21** | **22** |
| --- | --- | --- | --- | --- | --- | --- | --- | --- | --- | --- | --- | --- | --- | --- | --- | --- | --- | --- | --- | --- | --- | --- |
| *Mesocyparis. borealis* | 2 | 0 | 1 | 0 | 1 | 1 | 0 | 1 | 1 | 0 | 1 | 0 | 1 | 1 | 1 | 0 | 3 | 2 | 1 | 0 | 0 | 1 |
| *Mesocyparis. umbonata* | 1 | 0 | 0 | 0 | 1 | 1 | 0 | 1 | 0 | 0 | 0 | 0 | 0 | 0 | - | 0 | 1 | 0 | 1 | 1 | 0 | 1 |
| *Mesocyparis. beringiana* | 1 | 0 | 0 | 0 | 1 | 1 | 1 | 1 | 2 | 1 | 0 | 1 | 1 | 1 | 0 | 0 | 0 | 0 | 0 | 0 | 0 | 1 |
| *Mesocyparis. rosanovii* | 12 | 0 | 1 | 0 | 1 | 1 | 1 | 1 | 2 | 1 | 0 | 1 | 1 | 1 | 0 | 0 | 2 | 1 | 2 | 1 | 0 | 1 |
| *Mesocyparis. sinica* | 2 | 0 | 1 | 1 | 1 | 1 | 1 | 1 | 2 | 1 | 0 | 1 | 1 | 1 | 0 | 0 | 2 | 1 | 2 | 2 | 0 | 1 |
| *Juniperus. monticola* | 0 | 1 | 1 | 2 | 2 | 1 | 0 | 0 | - | - | - | - | - | 0 | - | 1 | 4 | 3 | 2 | 3 | 1 | 0 |
| *Xanthocyparis. ietmamensis* | 0 | 01 | 1 | 0 | 0 | 2 | 1 | 1 | 1 | 1 | 0 | 1 | 1 | 1 | 1 | 1 | 4 | 3 | 2 | 3 | 1 | 0 |
| *Hesperocyparis. arizonica* | 0 | 2 | 1 | 2 | 0 | 1 | 0 | 0 | - | - | - | - | - | 1 | 1 | 1 | 4 | 3 | 2 | - | 1 | 1 |

"-" indicates inapplicable.

**Note: Morphological characters and character states of Table S2**

Morphological data are obtained from based on our own observations and the literature [18–22].

1 Seed cone arrangement: decussate (0); spiral (1); whorled (2)

2 Number of ovuliferous cone scales: 4 (0); 6 (1); ≥ 8 (2)

3 Sizes of two pairs of cone scales: no (0); yes (1)

4 Shape of conescale's tip: acute (0); acuminate (1)

5 Mature conescale: free (0); apically connate (1); all fused (2)

6 Mature conescale: foliate (0); peltate (1); valvate (2)

7 Maximum angle between axis and shootlet on foliage branch: > 50° (0); ≤ 45° (1)

8 Leaves on foliage branch dimorphic: no (0); yes (1)

9 Shootlet's lateral leaves length/ width: < 3:1 (0); 3.5:1–5:1 (1); > 6:1 (2)

10 Shoot's lateral leaves length/ width: > 5.5:1 (0); 3:1–5:1 (1)

11 Lateral leaves on shootlet overlap facial leaves in the same ring: no (0); yes (1)

12 Facial leaves on shootlet overlap: no (0); yes (1)

13 Lateral leaves on shootlet overlap: no (0); yes (1)

14 Umbo position: near apex (0); in center (1)

15 Umbo near apex reflexed: no (0); yes (1)

16 Initial phyllotaxis on seedling: decussate (0); whorl (1)

17 Shootlet's facial leaves length (mean value) (mm): < 1.0 (0); 1.2–1.4 (1); 1.5–1.8 (2); > 2 (3)

18 Shootlet's lateral leaves length (mean value) (mm): ≤ 0.9 (0); 1.0–1.4 (1); ≥ 1.6 (2)

19 Seed cone length (mean value) (mm): ≤ 3.0 (0); 3.5–4.0 (1); > 4.2 (2)

20 Seed cone length/ width (mean value) (mm): ≤ 3.0 (0); 3.5–4.0 (1); 4.5–5.0 (2); > 5.5 (3)

21 Leaf gland: absent (0); present (1)

22 Leaf margin: denticulate (0); entire (1)
