# Supplementary material for: Fossil evidence reveals how plants responded to cooling during the Cretaceous-Paleogene transition
Source: BMC Plant Biol. 2019 Sep 13;19:402. doi: 10.1186/s12870-019-1980-y (PMC6743113; doi:10.1186/s12870-019-1980-y)
Supplement: Supplementary file 4 — Table S3. The seed cones sizes of Cupressus, their geographic information and mean annual temperature (MAT) of their habitats. (DOCX 19 kb) [file 12870_2019_1980_MOESM4_ESM.docx]

**Additional file 4.**

**Table S3.** The seed cones sizes of *Cupressus*, their geographic information and mean annual temperature (MAT) of their habitats

| **Species** | **Locality** | **Latitude** | **Longitude** | **Elevation** | **MAT** | **Dia.** | **Specimens’ No.** |
| --- | --- | --- | --- | --- | --- | --- | --- |
| *C. chengiana* | Mangkang | 29.6804 | 98.5866 | 4174 | 3.0 | 1.75 | 00012999 |
| *C. chengiana* | Xigu | 34.0509 | 104.396 | 2754 | 3.4 | 1.65 | 00013001 |
| *C. chengiana* | Xigu | 34.0509 | 104.396 | 2754 | 3.4 | 1.93 | 00013002 |
| *C. chengiana* | Kangding | 30.0525 | 101.958 | 4184 | 0.7 | 0.93 | 00013014 |
| *C. chengiana* | Xiaojin | 32.8991 | 101.706 | 4017 | -1.5 | 1.54 | 00013015 |
| *C. chengiana* | Jinchuan | 31.4868 | 102.057 | 3144 | 7.2 | 1.05 | 00013017 |
| *C. chengiana* | Kangding | 30.0525 | 101.958 | 4184 | 0.7 | 1.25 | 00047320 |
| *C. chengiana* | Maerkang | 31.911 | 102.234 | 3894 | 1.4 | 1.50 | 01057299 |
| *C. chengiana* | Maerkang | 31.911 | 102.234 | 3894 | 1.4 | 1.45 | 01523209 |
| *C. chengiana* | Lixian | 31.4542 | 103.164 | 4013 | 0.6 | 1.15 | 00013004 |
| *C. funebris* | Wulong | 29.3155 | 107.744 | 1096 | 13.5 | 0.96 | 00013143 |
| *C. funebris* | Shiqian | 27.522 | 108.232 | 554 | 16.8 | 1.01 | 00014308 |
| *C. funebris* | Xuanen | 29.9896 | 109.48 | 759 | 15.3 | 0.88 | 00012945 |
| *C. funebris* | Xinhuang | 27.3571 | 109.172 | 631 | 15.4 | 0.90 | 00012942 |
| *C. funebris* | Wenxian | 32.948 | 104.678 | 1900 | 10.2 | 1.21 | 01826392 |
| *C. funebris* | Wuning | 29.2625 | 115.101 | 275 | 16.4 | 0.99 | 00012912 |
| *C. funebris* | Anlong | 25.1153 | 105.467 | 1269 | 16.6 | 0.82 | 00014257 |
| *C. funebris* | Kunming | 25.0438 | 102.705 | 2181 | 13.6 | 1.05 | 00014324 |
| *C. funebris* | Nanzheng | 33.0049 | 106.931 | 973 | 12.7 | 1.12 | 00013107 |
| *C. funebris* | Jingdong | 24.4521 | 100.836 | 1940 | 15.5 | 1.33 | 01812400 |
| *C. duclouxiana* | Wudu | 33.3948 | 104.929 | 2352 | 6.7 | 1.53 | 01561030 |
| *C. duclouxiana* | Dali | 25.5895 | 100.226 | 2114 | 14.7 | 1.07 | 02041073 |
| *C. duclouxiana* | Kunming | 25.0438 | 102.705 | 2181 | 13.6 | 1.40 | 01458115 |
| *C. duclouxiana* | Daocheng | 29.0367 | 100.297 | 4620 | -0.3 | 1.27 | 00013024 |
| *C. duclouxiana* | Zhongdian | 27.8205 | 99.7074 | 3572 | 6.1 | 0.84 | 00053438 |
| *C. duclouxiana* | Xiaojin | 32.8991 | 101.706 | 4017 | -1.5 | 1.61 | 00063412 |
| *C. duclouxiana* | Dechang | 27.4054 | 102.173 | 2407 | 12.4 | 1.70 | 00063420 |
| *C. duclouxiana* | Lijiang | 26.8746 | 100.236 | 3409 | 7.3 | 1.95 | 00206327 |
| *C. duclouxiana* | Binchuan | 25.8303 | 100.578 | 1711 | 17.4 | 2.24 | 00013083 |
| *C. duclouxiana* | Chuxiong | 25.0331 | 101.544 | 1927 | 15.6 | 2.78 | 00982738 |
| *C. torulosa* | Deqin | 28.4912 | 98.9147 | 4032 | 3.2 | 1.73 | 01057309 |
| *C. torulosa* | Deqin | 28.4912 | 98.9147 | 4032 | 3.2 | 1.55 | 01458132 |
| *C. torulosa* | Bomi | 29.8648 | 95.745 | 4165 | 3.2 | 1.40 | 00042979 |
| *C. torulosa* | Bomi | 29.8648 | 95.745 | 4165 | 3.2 | 1.32 | 00053435 |
| *C. torulosa* | Langxian | 29.04812 | 93.0777 | 4880 | -1.4 | 1.71 | 00026747 |
| *C. torulosa* | Langxian | 29.04812 | 93.0777 | 4880 | -1.4 | 1.74 | 00026748 |
| *C. torulosa* | Motuo | 29.32274 | 95.3292 | 2815 | 10.8 | 1.35 | 00026755 |
| *C. torulosa* | Linzhi | 29.5803 | 94.4842 | 4353 | 1.8 | 1.29 | 00026757 |
| *C. gigantea* | Langxian | 29.04812 | 93.0777 | 4880 | -1.4 | 1.65 | 01458111 |
| *C. gigantea* | Milin | 29.2143 | 94.1996 | 4385 | 1.7 | 1.85 | 00042976 |
| *C. gigantea* | Linzhi | 29.5803 | 94.4842 | 4353 | 1.8 | 1.78 | 00026746 |
| *C. gigantea* | Linzhi | 29.5841 | 94.4843 | 4353 | 1.8 | 2.00 | 00063413 |
| *C. gigantea* | Linzhi | 29.5841 | 94.4843 | 4353 | 1.8 | 1.87 | 00063426 |
| *C. gigantea* | Milin | 29.2143 | 94.1996 | 4385 | 1.7 | 1.65 | 00017910 |
| *C. gigantea* | Langxian | 29.04812 | 93.0777 | 4880 | -1.4 | 1.63 | 00982904 |

The 45 samples of five *Cupressus* species (*C. chengiana*, *C. funebris*, *C. duclouxiana*, *C. torulosa*, and *C. gigantea*) are from PE Herbarium, which have seed cones and entire information on locality and elevation. The diameters (Dia.) of seed cones were measured to represent their seed cone sizes. Mean annual temperature (MAT) used the climate data set of (New *et al.* [51]) and then corrected to altitude of the plant site using a temperature lapse rate of 5 °C /km.
