# Supplementary material for: Fossil evidence reveals how plants responded to cooling during the Cretaceous-Paleogene transition
Source: BMC Plant Biol. 2019 Sep 13;19:402. doi: 10.1186/s12870-019-1980-y (PMC6743113; doi:10.1186/s12870-019-1980-y)
Supplement: Supplementary file 5 — Table S4. Fossil localities of Mesocyparis and the estimates of their paleo-latitudes and palaeo-longitudes. (DOCX 15 kb) [file 12870_2019_1980_MOESM5_ESM.docx]

**Additional file 5**

**Table S4.** Fossil localities of *Mesocyparis* and the estimates of their paleo-latitudes and palaeo-longitudes

| **Time** | **Age** | **Locality** | **Latitude** | **Longitude** | **Paleo-latitude** | **Paleo-longitude** |
| --- | --- | --- | --- | --- | --- | --- |
| Paleocene | Early to Middle Paleocene | Canada | 49.50 | -109.02 | 55.42 | -86.16 |
| Paleocene | Early Paleocene | Cananda | 53.09 | -116.68 | 60.08 | -93.20 |
| Paleocene | Early to Middle Paleocene | Cananda | 64.46 | -125.56 | 72.28 | -95.88 |
| Paleocene | Early Paleocene | Cananda | 54.40 | -94.40 | 61.59 | -94.40 |
| Maastrichtian | ca. 70 MA | Cananda | 51.49 | -83.19 | 59.48 | -83.19 |
| Maastrichtian | ca. 67 MA | Cananda | 51.42 | -112.65 | 59.65 | -84.62 |
| Paleocene | Paleocene | USA | 49.00 | -122.00 | 54.55 | -98.02 |
| Paleocene | Paleocene | USA | 44.27 | -104.95 | 49.67 | -83.78 |
| Maastrichtian | Middle Maastrichtian | Russia | 62.75 | 178.83 | 59.48 | -83.19 |
| Maastrichtian | Middle Maastrichtian | Russia | 62.73 | 178.07 | 76.28 | -177.10 |
| Maastrichtian | Middle Maastrichtian | Russia | 62.77 | 179.17 | 76.32 | -177.10 |
| Paleocene | Early Paleocene | Russia | 49.31 | 130.21 | 52.32 | 119.90 |
| Paleocene | ca.64~61 MA | China | 49.23 | 129.47 | 53.32 | 119.90 |

The data are from this study and the literature [18–22].
