# Supplementary material for: Fossil evidence reveals how plants responded to cooling during the Cretaceous-Paleogene transition
Source: BMC Plant Biol. 2019 Sep 13;19:402. doi: 10.1186/s12870-019-1980-y (PMC6743113; doi:10.1186/s12870-019-1980-y)
Supplement: Supplementary file 6 — Table S5. Fossil localities of Metasequoia and the estimates of their paleo-latitudes and palaeo-longitudes. (DOCX 20 kb) [file 12870_2019_1980_MOESM6_ESM.docx]

**Additional file 6**

**Table S5.** Fossil localities of *Metasequoia* and the estimates of their paleo-latitudes and palaeo-longitudes

| **Time** | **Locality** | **Latitude** | **Longitude** | **Paleo-latitude** | **Paleo-longitude** |
| --- | --- | --- | --- | --- | --- |
| Paleocene | USA | 42.84 | -105.77 | 48.41 | -85.16 |
| Paleocene | USA | 43.84 | -108.52 | 49.83 | -87.80 |
| Paleocene | USA | 43.85 | -110.52 | 50.15 | -89.99 |
| Paleocene | USA | 43.91 | -107.55 | 49.74 | -86.72 |
| Paleocene | USA | 43.92 | -108.51 | 49.90 | -87.76 |
| Paleocene | USA | 44.25 | -108.80 | 50.27 | -87.96 |
| Paleocene | USA | 44.26 | -108.81 | 50.28 | -87.97 |
| Paleocene | USA | 44.67 | -107.00 | 50.39 | -85.85 |
| Paleocene | USA | 45.37 | -105.55 | 50.84 | -84.03 |
| Paleocene | USA | 45.50 | -102.00 | 50.37 | -80.17 |
| Paleocene | USA | 45.63 | -106.26 | 51.21 | -84.70 |
| Paleocene | USA | 46.17 | -105.62 | 51.63 | -83.80 |
| Paleocene | USA | 46.18 | -108.44 | 52.09 | -86.87 |
| Paleocene | USA | 46.23 | -108.43 | 52.14 | -86.84 |
| Paleocene | USA | 46.87 | -105.44 | 52.28 | -83.34 |
| Paleocene | USA | 46.91 | -103.52 | 52.00 | -81.25 |
| Paleocene | USA | 46.92 | -103.82 | 52.06 | -81.59 |
| Paleocene | USA | 46~48 | -101~-103 | 51.83 | -79.58 |
| Paleocene | USA | 47.37 | -104.40 | 52.59 | -82.01 |
| Paleocene | USA | 47.47 | -104.50 | 52.70 | -82.08 |
| Paleocene | USA | 47.50 | -122.12 | 53.07 | -98.51 |
| Paleocene | USA | 47.58 | -102.15 | 52.41 | -79.50 |
| Paleocene | USA | 47.00 | -104.00 | 52.16 | -81.73 |
| Paleocene | Russia | 47.00 | 143.00 | 51.37 | 134.12 |
| Paleocene | USA | 48.18 | -110.11 | 54.31 | -87.93 |
| Paleocene | USA | 48.41 | -109.28 | 54.40 | -86.92 |
| Paleocene | Canada | 48.56 | -100.41 | 53.06 | -77.23 |
| Paleocene | USA | 48.75 | -106.25 | 54.23 | -83.44 |
| Paleocene | USA | 48.88 | -122.50 | 54.79 | -102.39 |
| Paleocene | USA | 48.99 | -105.23 | 54.30 | -82.23 |
| Paleocene | Canada | 50.34 | -105.04 | 55.57 | -81.41 |
| Paleocene | Canada | 50.40 | -125.50 | 56.76 | -105.01 |
| Paleocene | Canada | 53.35 | -114.40 | 59.99 | -90.41 |
| Paleocene | Canada | 54.31 | -118.69 | 61.56 | -94.92 |
| Paleocene | Canada | 54.50 | -102.00 | 59.04 | -75.95 |
| Paleocene | Canada | 57.50 | -131.75 | 64.62 | -107.89 |
| Paleocene | USA | 61.64 | -148.96 | 69.20 | -120.86 |
| Paleocene | USA | 62.8–63.8 | -148.7–-152.3 | 73.57 | -130.06 |
| Paleocene | Russia | 62.00 | 164.00 | 71.92 | 160.10 |
| Paleocene | USA | 63.50 | -107.50 | 68.46 | -74.92 |
| Paleocene | Spitsbergen | 78.50 | 18.50 | 72.77 | 11.81 |
| Paleocene | Scotland | 57.37 | -6.25 | 51.65 | -7.12 |
| Paleocene | Scotland | 59.23 | -2.53 | 53.45 | -3.82 |
| Paleocene | China | 34.50 | 110.81 | 34.93 | 102.81 |
| Paleocene | USA | 46.41 | -105.87 | 51.90 | -83.98 |
| Paleocene | USA | 48.94 | -122.82 | 54.90 | -102.72 |
| Paleocene | Canada | 54.00 | -123.50 | 59.88 | -100.66 |
| Paleocene | USA | 56.18 | -158.27 | 67.42 | -140.00 |
| Paleocene | USA | 61.31 | -149.19 | 69.01 | -121.68 |
| Paleocene | USA | 61.43 | -148.59 | 68.92 | -120.85 |
| Paleocene | USA | 78.75 | 13.00 | 72.96 | 8.08 |
| Paleocene | USA | 58.19 | -154.11 | 67.95 | -132.17 |
| Paleocene | USA | 59.20 | -151.51 | 68.02 | -127.67 |
| Paleocene | USA | 62.55 | -164.00 | 73.36 | -149.16 |
| Paleocene | Kazakhstan | 51.11 | 52.52 | 46.95 | 45.37 |
| Paleocene | Siberia | 51.00 | 142.00 | 55.22 | 132.04 |
| Paleocene | USA | 42.28 | -108.10 | 48.24 | -87.86 |
| Paleocene | USA | 47.00 | -101.00 | 51.65 | -78.51 |
| Paleocene | Greenland | 82.00 | -19.00 | 70.94 | 5.43 |
| Paleocene | USA | 48.67 | -122.67 | 54.61 | -102.67 |
| Paleocene | China | 48.75 | 128.31 | 51.55 | 118.06 |
| Maastrichtian –Paleocene | USA | 48.00 | -120.20 | 53.54 | -100.10 |
| Maastrichtian –Paleocene | Canada | 54.93 | -122.65 | 62.72 | -99.36 |
| Maastrichtian | USA | 48.67 | -120.42 | 54.23 | -100.04 |
| Maastrichtian | Russia | 62.50 | 175.00 | 73.32 | 176.73 |
| Cretaceous | Russia | 50.40 | 51.25 | 43.30 | 44.37 |
| Cretaceous | Russia | 51.00 | 117.00 | 51.13 | 104.19 |
| Cretaceous | Russia | 52.48 | 79.47 | 47.78 | 68.79 |
| Cretaceous | Russia | 54.00 | 132.00 | 56.14 | 118.03 |
| Cretaceous | Russia | 56.00 | 158.00 | 67.37 | 152.92 |
| Cretaceous | Russia | 60.68 | 125.00 | 61.61 | 107.60 |
| Cretaceous | Russia | 60.00 | 148.00 | 64.09 | 132.03 |
| Cretaceous | Russia | 65.00 | 175.50 | 78.23 | 174.18 |
| Cretaceous | Russia | 67.50 | 175.00 | 77.56 | 162.00 |
| Cretaceous | Russia | 68.50 | 158.35 | 73.52 | 136.98 |
| Cretaceous | Japan | 35.51 | 136.21 | 43.68 | 124.22 |
| Cretaceous | USA | 39.17 | -108.14 | 46.77 | -83.81 |
| Cretaceous | Japan | 40.14 | 141.43 | 49.31 | 127.97 |
| Cretaceous | USA | 41.59 | -109.23 | 49.32 | -84.10 |
| Cretaceous | USA | 41.68 | -108.79 | 49.32 | -83.58 |
| Cretaceous | USA | 41.83 | -106.17 | 48.95 | -80.63 |
| Cretaceous | China | 42.88 | 130.30 | 44.24 | 118.11 |
| Cretaceous | USA | 43.03 | -104.65 | 49.80 | -78.47 |
| Cretaceous | USA | 44.31 | -112.36 | 52.54 | -86.49 |
| Cretaceous | USA | 46.30 | -103.90 | 52.78 | -76.15 |
| Cretaceous | USA | 46.00 | -112.50 | 54.19 | -85.90 |
| Cretaceous | USA | 47.73 | -109.65 | 55.31 | -81.81 |
| Cretaceous | Canada | 49.08 | -123.58 | 56.18 | -99.08 |
| Cretaceous | Russia | 50.00 | 130.00 | 51.82 | 115.77 |
| Cretaceous | Canada | 54.00 | -123.00 | 60.65 | -94.93 |
| Cretaceous | Denmark | 61.80 | -5.75 | 61.80 | -5.75 |
| Cretaceous | Russia | 63~65 | 175~179 | 78.19 | 173.16 |
| Cretaceous | Siberia | 63.00 | 124.00 | 63.69 | 105.12 |
| Cretaceous | Siberia | 63.00 | 125.00 | 63.83 | 106.10 |
| Cretaceous | Siberia | 63.00 | 148.00 | 66.98 | 130.07 |
| Cretaceous | Siberia | 64.00 | 124.00 | 64.64 | 104.40 |
| Cretaceous | Siberia | 65.00 | 125.00 | 65.73 | 104.60 |

The data are from Liu *et al.* [17].
