# Supplementary material for: Fossil evidence reveals how plants responded to cooling during the Cretaceous-Paleogene transition
Source: BMC Plant Biol. 2019 Sep 13;19:402. doi: 10.1186/s12870-019-1980-y (PMC6743113; doi:10.1186/s12870-019-1980-y)
Supplement: Supplementary file 7 — Table S6. Fossil localities of Nordenskioldia and the estimates of their paleo-latitudes and paleo-longitudes. (DOCX 16 kb) [file 12870_2019_1980_MOESM7_ESM.docx]

**Additional file 7**

**Table S6.** Fossil localities of *Nordenskioldia* and the estimates of their paleo-latitudes and paleo-longitudes

| **Time** | **Locality** | **Latitude** | **Longitude** | **Paleo-latitude** | **Paleo-longitude** |
| --- | --- | --- | --- | --- | --- |
| Paleocene | Russia | 52.33 | 87.42 | 51.14 | 77.73 |
| Cretaceous | Russia | 74.60 | 148.50 | 77.73 | 114.97 |
| Paleocene | Russia | 49.10 | 130.75 | 52.17 | 120.52 |
| Maastrichtian –Danian | Russia | 64.40 | 176.05 | 77.68 | 175.81 |
| Cretaceous | Russia | 61.00 | 124.50 | 61.85 | 106.92 |
| Paleocene | Kazakhstan | 43.85 | 77.23 | 41.77 | 69.74 |
| Paleocene | Kazakhstan | 47.70 | 84.20 | 46.24 | 75.71 |
| Paleocene | Japan | 43.00 | 142.00 | 47.31 | 133.89 |
| Paleocene | Japan | 40.20 | 141.72 | 49.98 | 131.88 |
| Cretaceous | China | 49.00 | 130.05 | 50.86 | 116.21 |
| Paleocene | China | 47.52 | 87.08 | 46.34 | 78.53 |
| Paleocene | China | 49.00 | 130.05 | 51.99 | 119.81 |
| Paleocene | Mongolia | 43.87 | 109.12 | 44.50 | 99.74 |
| Paleocene | Spitsbergen | 78.08 | 14.00 | 72.30 | 8.86 |
| Paleocene | Spitsbergen | 78.10 | 14.20 | 72.32 | 8.99 |
| Paleocene | Greenland | 70.58 | -53.17 | 62.92 | -23.03 |
| Paleocene | Greenland | 70.00 | -52.00 | 62.24 | -22.74 |
| Paleocene | USA | 46.10 | -109.98 | 52.26 | -88.60 |
| Paleocene | USA | 45.33 | -106.50 | 50.96 | -85.07 |
| Paleocene | USA | 45.10 | 107.00 | 45.46 | 97.34 |
| Paleocene | USA | 46.00 | -112.50 | 52.56 | -91.43 |
| Paleocene | USA | 46.72 | -101.50 | 51.47 | -79.16 |
| Paleocene | USA | 44.82 | -107.00 | 50.54 | -85.80 |
| Paleocene | USA | 41.27 | -109.20 | 47.42 | -89.37 |
| Paleocene | USA | 46.00 | -112.50 | 52.56 | -91.43 |
| Paleocene | USA | 60.82 | -148.90 | 68.54 | -122.23 |
| Paleocene | USA | 56.82 | -158.80 | 68.12 | -139.83 |
| Paleocene | USA | 55.82 | -159.57 | 67.46 | -142.11 |
| Paleocene | USA | 48.00 | -120.50 | 53.59 | -100.43 |
| Paleocene | Canada | 53.35 | -112.40 | 59.67 | -88.12 |
| Paleocene | Canada | 64.92 | -125.48 | 72.69 | -95.26 |
| Maastrichtian | Canada | 51.50 | -112.50 | 59.46 | -82.96 |

The data are from Wang *et al.* [52].
